# Self‐Propelled HPB@Lip@AB Nanomotors Ameliorate Dry Eye Disease

**DOI:** 10.1002/advs.77681

**Published:** 2026-09-22

**Authors:** Jing Li, Xi Wan, Xinru Su, Zechuan Li, Yue Wang, Ying Wang, Ruofei Li, Huangzhao Wei, Jianbin Zhang, Shuai Wang

**Affiliations:** ^1^ Department of Ophthalmology Second Affiliated Hospital of Dalian Medical University Dalian China; ^2^ Institute of Heart and Vascular Diseases Second Affiliated Hospital of Dalian Medical University Dalian China; ^3^ Dalian Institute of Chemical Physics Chinese Academy of Sciences Dalian China; ^4^ College of Pharmacy Dalian Medical University Dalian China

**Keywords:** drug delivery, dry eye disease, hydrogen, inflammation, mitochondrial dysfunction, nanomotor

## Abstract

Dry eye disease (DED) is a common ocular surface disorder closely associated with mitochondrial dysfunction and excessive accumulation of reactive oxygen species (ROS). However, existing treatments are limited by ocular surface barriers. This study developed an integrated nanomotor‐based eye drop system (HPB@Lip@AB) combining self‐propulsion, mitochondrial repair, and multiple antioxidant functions. The nanomotor consists of a hollow Prussian blue (HPB) nanozyme core loaded with ammonia borane (AB) as a molecular hydrogen (H_2_) precursor, encapsulated within a liposomal shell to form a core–shell structure. In the DED microenvironment, HPB catalyzes the decomposition of hydrogen peroxide (H_2_O_2_) to generate oxygen bubbles, propelling the nanomotors to rapidly penetrate the tear film barrier. Following cellular uptake, AB is proposed to generate H_2_ in acidic intracellular environments, potentially acting synergistically with HPB to exert antioxidant, anti‐apoptotic, and anti‐inflammatory effects. This restores tear secretion, stabilizes the tear film, and repairs the corneal epithelium. H_2_‐related effects were associated with increased aldehyde dehydrogenase 1 family member L2 (ALDH1L2) expression, improved mitochondrial function, and reduced apoptosis and inflammation. Our nanomotor strategy enables efficient DED treatment through combined mechanisms, offering a promising platform for high‐penetration ocular drug delivery.

## Graphical abstract

A self‐propelled HPB@Lip@AB nanomotor system is developed for dry eye disease (DED) therapy, facilitating rapid penetration across ocular surface barriers while integrating hydrogen therapy with multi‐enzyme‐like antioxidant activity. The nanoplatform restores mitochondrial function, suppresses oxidative stress, inflammation, and apoptosis, and provides a multifunctional nanotherapeutic strategy for comprehensive and effective DED management.

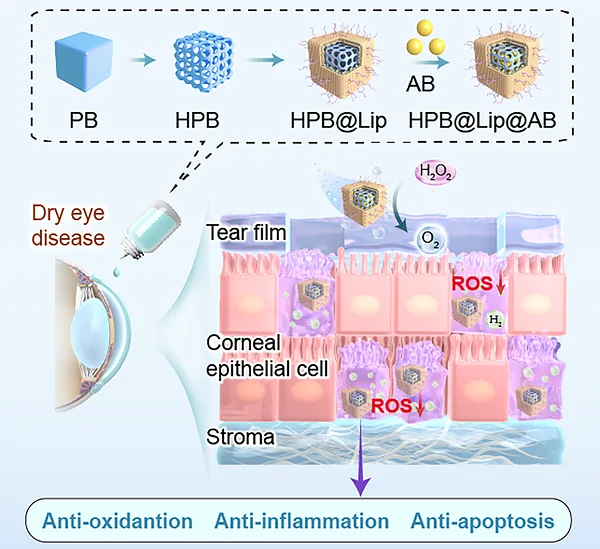

## 1 Introduction

Dry eye disease (DED) is a prevalent ocular disorder, affecting approximately 30% of the global population. It is associated with tear film instability, ocular discomfort, visual fatigue, and even vision impairment in severe cases [1, 2, 3]. The key pathological features include reduced tear secretion, excessive evaporation, altered tear composition, and epithelial damage accompanied by inflammation [4, 5, 6, 7]. Current clinical management of DED mainly relies on artificial tears and topical anti‐inflammatory agents, such as corticosteroids and cyclosporine A (CsA) [8, 9, 10]. However, their benefits are often transient and accompanied by side effects that limit patient compliance. Therefore, developing safe and effective therapies that directly target the core pathogenic mechanisms of DED has become an important focus of research. Mounting evidence has indicated that mitochondrial dysfunction is a central contributor to DED pathogenesis [11, 12]. Following mitochondrial injury, overproduction of reactive oxygen species (ROS), such as hydrogen peroxide (H_2_O_2_), hydroxyl radicals (·OH), and superoxide anions (·O_2_^−^) can induce corneal epithelial inflammation and apoptosis, promoting DED progression [13, 14]. Thus, scavenging excessive ROS represents a promising therapeutic approach for treating DED.

One of the most promising ways of scavenging ROS is hydrogen therapy. Molecular hydrogen (H_2_), characterized by its tiny size, electrical neutrality, and non‐polarity, exhibits excellent biocompatibility and tissue penetration. It can freely pass through cell membranes and reach key areas such as mitochondria to clear ROS, especially hydroxyl radicals (·OH) and peroxynitrite (ONOO^−^) [15, 16]. This scavenging effect can effectively inhibit oxidative damage, alleviate inflammation, and prevent apoptosis [17, 18]. H_2_ has been found to ameliorate retinal branch vein occlusion [19], age‐related macular degeneration [20], and diabetic retinopathy [21]. Additionally, studies have shown that hydrogen‐rich saline can improve DED in rats [22]. However, H_2_’s poor solubility and rapid evaporation in water severely limit the treatment efficacy. Accordingly, hydrogen storage materials such as ammonia borane (AB) have recently been investigated as delivery platforms. AB is a typical H_2_ precursor with considerable H_2_ storage density and can decompose to produce H_2_ gas in an acid‐dependent manner [23, 24, 25]. It has been used to ameliorate osteoarthritis inflammation [26] and promote healing of infected diabetic wounds [27].

In recent years, artificial nanozymes have been proven to be another promising technology for ROS scavenging. Nanozymes could mimic the activities of natural enzymes such as superoxide dismutase (SOD), peroxidase (POD), and catalase (CAT) to efficiently neutralize ROS, with the advantages of low cost and high stability [28]. In the treatment of DED, nanozyme eye drops including CeO_2_, Ce‐MOF, PBnZ, and FeMn‐DAN have been developed with excellent therapeutic effect [29, 30, 31]. Prussian blue (PB), an FDA‐approved antidote for radioactive cesium‐137 and thallium poisoning, could mimic multiple enzyme‐like activities (notably SOD, CAT, and POD) to alleviate the inflammatory responses of various diseases, such as osteoarthritis, inflammatory bowel disease, and pancreatitis [32, 33, 34]. Additionally, hollow Prussian blue (HPB) has been widely applied in drug delivery due to its good biocompatibility and stability. Liposomes are representative lipid‐based delivery platforms with biomimetic structures similar to cellular membranes, providing favorable biocompatibility, biodegradability, and cargo‐delivery capacity [35, 36]. They can protect encapsulated components from premature degradation, improve their stability, and facilitate delivery to target sites.

Despite the excellent therapeutic effect of DED, both H_2_ therapy and nanozyme‐based strategies still have to face the ocular surface barriers, such as limited corneal permeability, constant blinking, and turnover of tears [37, 38]. Prolonging retention time and increasing the permeability through ocular tissue, mainly the cornea, are the two main strategies used in topical administration. In recent years, an emerging nanotechnology, the nanomotor, has been developed to overcome various physiologic barriers. Nanomotors, a class of nanodevices with self‐propelled motion capability, can obtain thrust from chemical reactions with environmental substances (H_2_O_2_, glucose, and urea) or from external stimuli (magnetic fields, ultrasound, light, and electrical fields) [39]. As H_2_O_2_ is commonly distributed in numerous diseases, H_2_O_2_‐powered nanomotors have been widely constructed by various nano‐sized structures with CAT‐like activities, including Pt, Au, Pd, MnO_2_, CeO_2_, and Prussian blue [40]. They convert H_2_O_2_ into water and O_2_, generating bubbles to propel their motion. As high levels of ROS (including H_2_O_2_) are present in the tear film of DED patients, the H_2_O_2_‐powered nanomotors may represent a promising strategy to overcome the ocular surface barriers.

**FIGURE 1:**
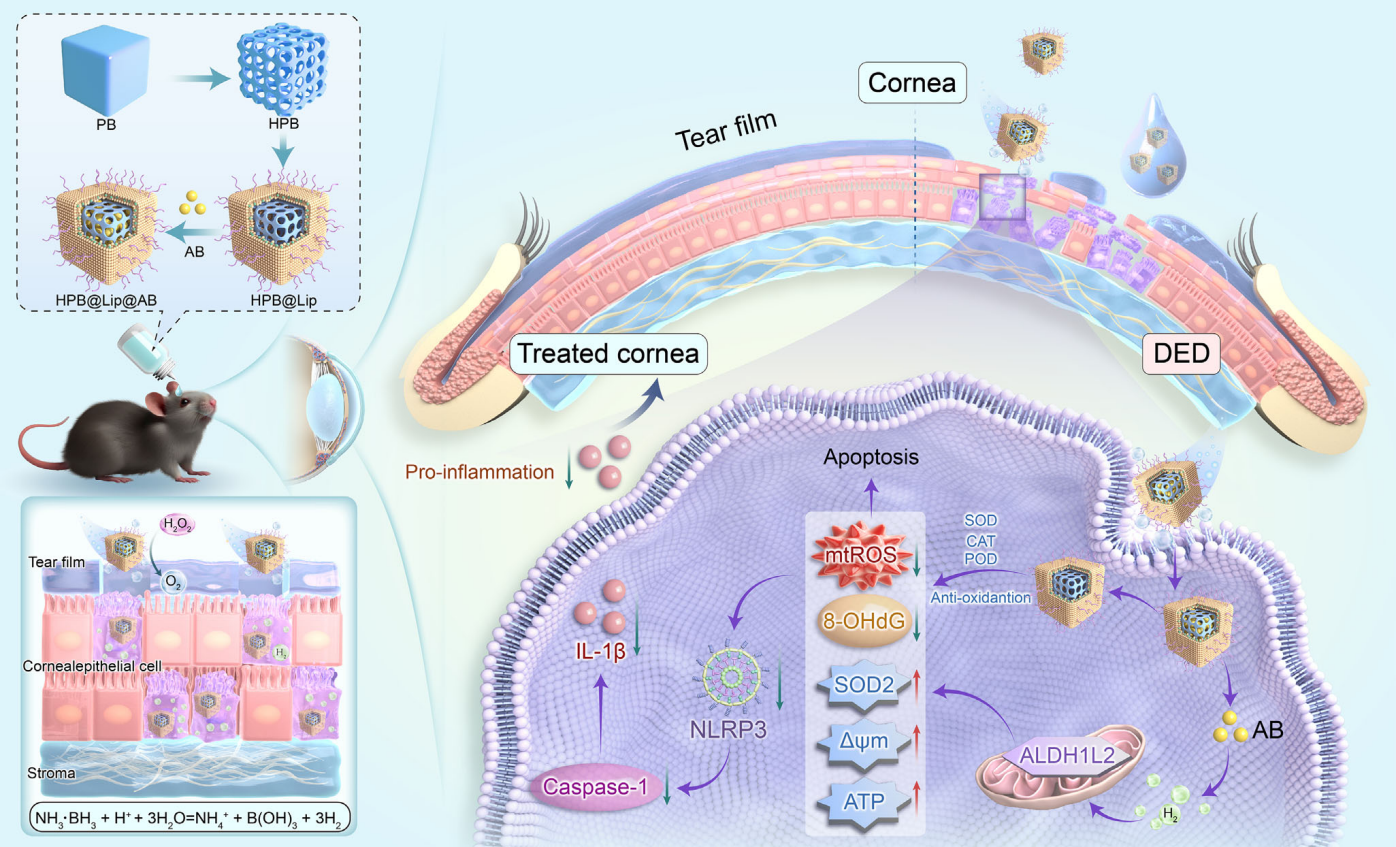
Schematic illustration of the preparation process of HPB@Lip@AB nanomotors and the potential mechanism in the treatment of DED.

Herein, we combine a triple‐strategy approach integrating hydrogen therapy, nanozyme catalysis, and nanomotor propulsion, to construct a multifunctional nanoplatform named HPB@Lip@AB for the treatment of DED. As shown in Figure 1, PB nanozyme was etched to form HPB, and then coated with a liposome to form HPB@Lip. Subsequently, the H_2_ donor AB was encapsulated to form HPB@Lip@AB nanomotors. In the treatment of DED, HPB@Lip@AB can be topically administered as eye drops, and HPB can catalyze the excessive H_2_O_2_ in tear film to generate oxygen bubbles. The directional ejection of these gas bubbles provides self‐propulsion, enabling the nanomotors to rapidly penetrate the tear film barrier and effectively accumulate in the corneal epithelium. In corneal epithelial cells, AB may diffuse out of the nanomotors and generate H_2_ in acidic intracellular environments. H_2_ and HPB act synergistically to scavenge excessive ROS, thereby conferring combined benefits in antioxidation, anti‐inflammation, and anti‐apoptosis. In vitro studies revealed that HPB@Lip@AB significantly improved mitochondrial ultrastructure and restored mitochondrial membrane potential (ΔΨm), adenosine triphosphate (ATP) content, and SOD2 levels, while concurrently reducing ROS/mitochondrial ROS (mtROS) and 8‐hydroxy‐2'‐deoxyguanosine (8‐OHdG) levels. In vivo studies revealed that HPB@Lip@AB significantly improved both tear secretion and tear film stability of DED mice, as well as restored their corneal structure and epithelial thickness. Transcriptomic analysis and reverse validation experiments suggested that increased aldehyde dehydrogenase 1 family member L2 (ALDH1L2) expression may contribute to the protective effects of HPB@Lip@AB, including its antioxidant effects, by jointly alleviating mitochondrial dysfunction, apoptosis, and inflammatory responses in DED. Taken together, HPB@Lip@AB represents a breakthrough nano‐eye‐drop strategy for DED, integrating corneal tissue penetration, mitochondrial repair, and multi‐pathway therapeutic synergy.

## 2 Results

### 2.1 Synthesis and Characterization of HPB@Lip@AB

**FIGURE 2:**
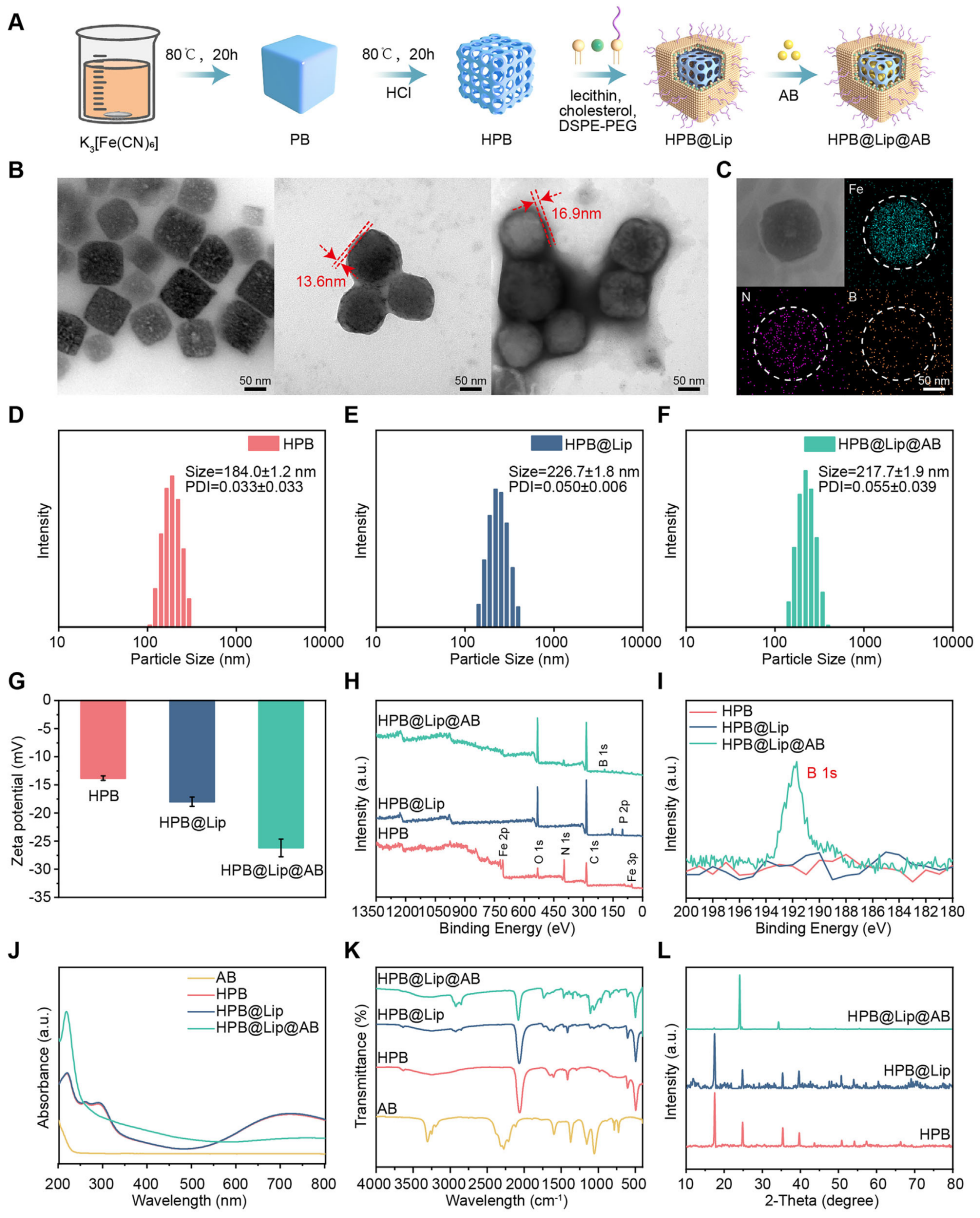
Fabrication and characterization of HPB@Lip@AB. (A) Schematic illustration of HPB@Lip@AB. (B) TEM images of HPB, HPB@Lip, and HPB@Lip@AB. (C) Elemental mapping of HPB@Lip@AB. (D–F) Particle size and polydispersity index (PDI) of HPB, HPB@Lip, and HPB@Lip@AB. (G) Zeta potentials of HPB, HPB@Lip, and HPB@Lip@AB (n = 3). (H) The XPS survey spectrum of HPB, HPB@Lip, and HPB@Lip@AB. (I) High‐resolution XPS spectra of the B 1s region for HPB, HPB@Lip, and HPB@Lip@AB. (J) UV–Vis absorption spectral profiles of AB, HPB, HPB@Lip, and HPB@Lip@AB. (K) FTIR analysis of AB, HPB, HPB@Lip, and HPB@Lip@AB. (L) XRD patterns of HPB, HPB@Lip, and HPB@Lip@AB.

The preparation process of HPB@Lip@AB was shown in Figure 2A. First, PB was synthesized through an acid‐induced decomposition of potassium ferricyanide (K_3_[Fe(CN)_6_]), and etched to form hollow‐structured HPB. Then, HPB was coated with a liposome to form HPB@Lip, which was then loaded with AB to form HPB@Lip@AB. The characterizations of HPB, HPB@Lip, and HPB@Lip@AB were subsequently carried out through transmission electron microscopy (TEM), dynamic light scattering (DLS), x‐ray photoelectron spectroscopy (XPS), ultraviolet‐visible spectroscopy (UV–Vis), Fourier‐transform infrared spectroscopy (FTIR), and x‐ray diffraction (XRD) methods. All three nanoparticles were easily dispersed in water and formed clear blue solutions (Figure S1). TEM images of HPB showed a uniform porous cubic morphology with a particle size of approximately 100 nm. After coating with liposome, the distinct core–shell structures were observed in both HPB@Lip and HPB@Lip@AB, with the outer shell thicknesses of 13.6 and 16.9 nm (Figure 2B). Elemental mapping of HPB@Lip@AB showed a uniform distribution of Fe, N, and B within the nanoparticle region (delineated by the white dashed circle), indicating that AB was successfully loaded into the nanoparticle (Figure 2C). Next, DLS results demonstrated that the hydrodynamic size distributions of HPB, HPB@Lip and HPB@Lip@AB were narrow, with the average sizes of 184.0, 226.7 and 217.7 nm (Figure 2D–F). Due to the presence of a hydration shell in aqueous solution, the sizes of DLS were larger than those of TEM. The zeta potential of HPB was −13.8 mV, which decreased to −18.0 mV (HPB@Lip) and −26.2 mV (HPB@Lip@AB) after coating with negative phospholipid (Figure 2G). The high zeta potential values could ensure their high colloidal stabilities. Furthermore, XPS was conducted to analyze the surface chemical compositions and the elemental states. The obvious peaks of Fe 3p (54.4 eV), C 1s (283.8 eV), O 1s (530.7 eV), and Fe 2p (707.7 eV of Fe 2p_3/2_ and 721.7 eV of Fe 2p_1/2_) could be observed in the survey spectrum and the high‐resolution spectra of HPB (Figure 2H and Figure S2A–F). After liposome coating, the peaks of N 1s, Fe 2p, and Fe 3p decreased and even disappeared. Instead, a P 2p peak (133.1 eV) appeared in the HPB@Lip and HPB@Lip@AB spectra. In particular, the new peak of B 1s (191.7 eV) in the HPB@Lip@AB confirmed the presence of AB (Figure 2I). Moreover, the UV–Vis absorption spectra revealed the characteristic absorption peaks of HPB at 218 and 719 nm. The absorbance of AB gradually decreased from 200 to 250 nm, with no obvious characteristic absorption peak (Figure 2J). The chemical groups of samples were further verified by FTIR. The characteristic absorption peaks of N─H stretching vibration (3305 cm^−1^), B─H stretching vibration (2276 cm^−1^), B─H bending vibration (1372 cm^−1^), and B‐N stretching vibration (1155 cm^−1^) were clearly identified in the AB spectrum. Similarly, Fe(II)─C≡N─Fe(III) bond (stretching vibration at 2060 cm^−1^) of HPB, C─H bond (stretching vibration at 2925 cm^−1^) and C═O (stretching vibration at 1737 cm^−1^) of lecithin could be observed.

The mentioned characteristic absorption peaks were also present in the HPB@Lip@AB spectrum, indicating the successful synthesis of the designed structure (Figure 2K). Furthermore, the crystal structures of samples were analyzed by XRD. The diffraction peaks of HPB at 2θ angles of 17.5° (200), 24.8° (220), 35.4° (400), and 39.7° (420) matched the standard data card of cubic Prussian blue (JCPDS No. 73–0687) [41]. The pattern was not changed after liposome coating and AB loading. In the XRD pattern of HPB@Lip@AB, the strong peak at 2θ angles of 24.1° further verified the successful embedding of AB (Figure 2L). Finally, we evaluated the colloidal stabilities of HPB, HPB@Lip, and HPB@Lip@AB in five different media‐water, PBS, simulated tear fluid (STF), DMEM, and DMEM + FBS, by monitoring changes in particle size via DLS over 48 h. They all maintained stable particle sizes at approximately 200 nm across all tested media (Figure S3A–C), indicating excellent colloidal stability without aggregation or precipitation. Taken together, these results provided conclusive evidence for the successful fabrication of HPB@Lip@AB with a core–shell structure.

### 2.2 In Vitro H_2_ Release

**FIGURE 3:**
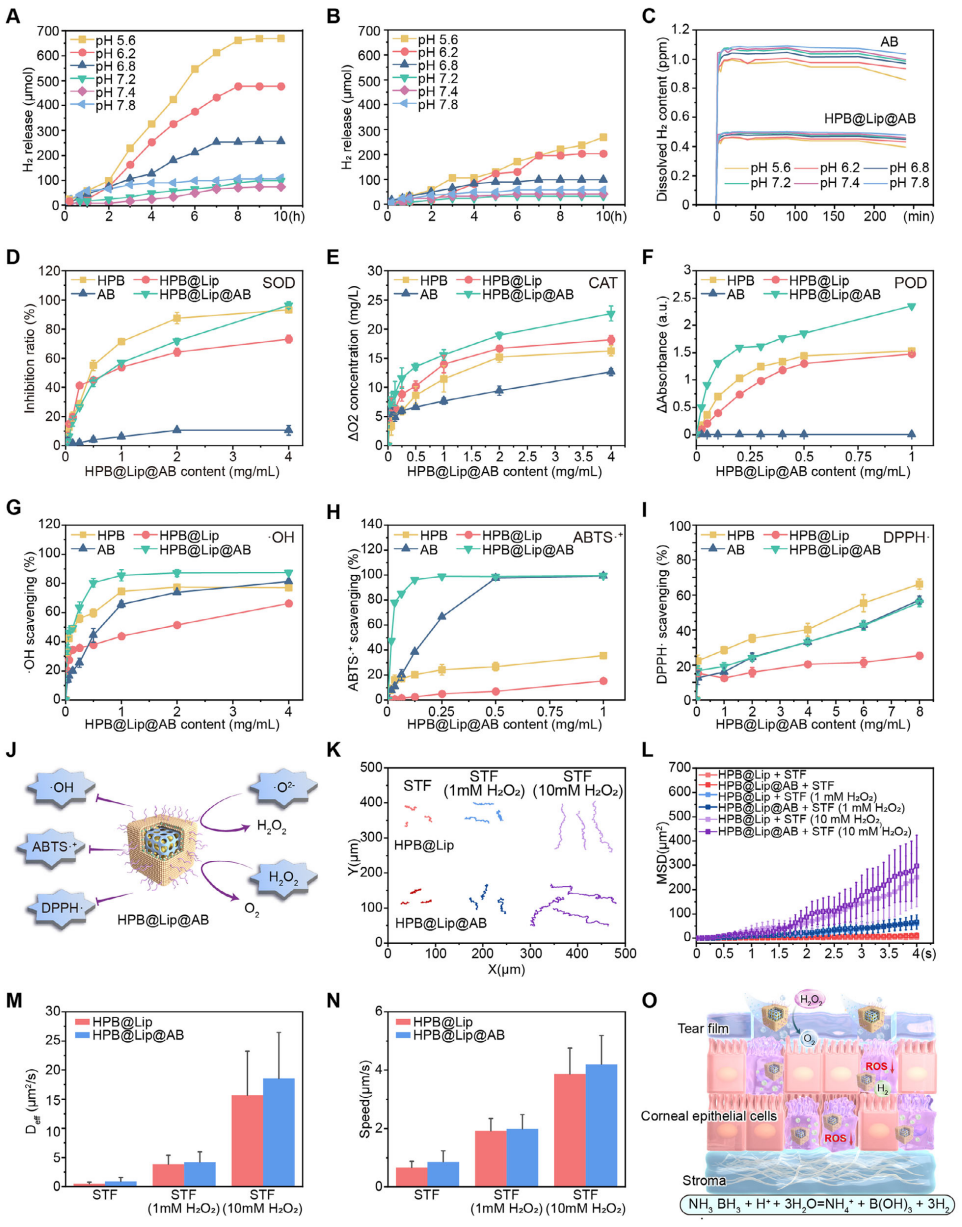
H_2_ release behavior, multienzyme‐like activity, antioxidant activity, and nanomotor motion behavior of HPB@Lip@AB. (A‐B) H_2_ release curves of AB and HPB@Lip@AB under different pH conditions. (C) The dissolved H_2_ contents in AB and HPB@Lip@AB solutions under different pH conditions. (D–F) The SOD‐like, CAT‐like, and POD‐like activities of HPB, HPB@Lip, AB, and HPB@Lip@AB (n = 3). (G–I) ·OH, ABTS·^+^ and DPPH· scavenging capacities of HPB, HPB@Lip, AB, and HPB@Lip@AB (n = 3). (J) Schematic illustration of the multi‐enzyme‐like and antioxidant activities of HPB@Lip@AB. (K‐N) The movements, MSD, *D*_eff_, and average speed of HPB@Lip and HPB@Lip@AB in STF with different concentrations of H_2_O_2_ (n = 15). (O) Schematic illustration of nanomotor motion behavior of HPB@Lip@AB and the mechanism for treating DED.

Generally, the hydrolysis of AB occurs in the presence of a catalyst, releasing three equivalents of H_2_ per mole of AB. Hence, the amount of AB could be quantified through the generated H_2_. Here, we used a Co‐Fe‐B catalyst to hydrolyze and measured the volume of H_2_ with the typical water displacement method. The loading capacity (LC) of AB in HPB@Lip@AB was calculated to be 10%–15% for different batches. Apart from the catalyst, H^+^ could also lead to the hydrolysis of AB with the reaction mechanism presented as BH_3_·NH_3_ + H^+^ + 3H_2_O = NH_4_^+^ + B(OH)_3_ + 3H_2_ [42]. As pH is an important physiological indicator, the effect of pH on the H_2_ release behavior of AB and HPB@Lip@AB was subsequently evaluated. In this work, the total amount of AB was maintained at 20 mg (648 µmol) in each group, and the volume of generated H_2_ was measured by the water‐filled gas burette and calculated according to Equation 1. The release of H_2_ from AB exhibited a typical acidic‐responsive behavior. In normal (pH = 7.2) and alkaline (pH = 7.4 and 7.8) conditions, limited H_2_ was generated from AB within 10 h (less than 100 µmol), with few H_2_ bubbles observed. However, abundant H_2_ was generated from AB in acidic conditions, reaching 670 µmol at pH 5.6 (Figure 3A). In comparison with AB, the amounts of H_2_ released from HPB@Lip@AB were significantly decreased (Figure 3B, Figure S4). This was because the encapsulated AB was protected against H^+^ by the liposome shell, and the release rate was slow. In addition, we detected the dissolved H_2_ contents in the solutions using a H_2_ detector. Figure 3C showed that the dissolved H_2_ content was maintained at approximately 1 ppm in AB solutions and 0.5 ppm in HPB@Lip@AB. It appeared that the dissolved H_2_ content was not influenced by pH and could be maintained for a long time (at least 4 h). To further characterize the intracellular behavior of HPB@Lip@AB, we examined its localization in corneal epithelial cells. Coumarin 6 (C6)‐labeled HPB@Lip@AB partially overlapped with lysosome‐associated membrane protein 1 (LAMP1), suggesting cellular internalization and partial association with lysosomal compartments (Figure S5). A clinical study demonstrated that the tear film pH values in DED patients and healthy persons were 7.46 ± 0.24 and 7.45 ± 0.23 [43]. These results suggest that the near‐neutral tear‐film environment may be less favorable for H_2_ generation, whereas acidic intracellular environments may favor H_2_ generation after cellular internalization.

### 2.3 Multienzyme‐Like Activities and Antioxidant Activities of HPB@Lip@AB

Excess ROS in the ocular tissues are a major culprit of DED. They can damage functional nucleic acids and proteins directly, as well as induce inflammation and tear hyperosmolarity. Hence, removing excess ROS is an effective strategy for relieving DED. In the structure of HPB@Lip@AB, HPB could eliminate ROS by exerting multienzyme‐like activities (notably SOD, CAT, and POD), while AB could produce H_2_ to directly reduce ROS. Here, we systematically studied the combined effects of HPB@Lip@AB in eliminating ROS through multiple mechanisms.

The SOD/CAT/POD‐like activities of HPB@Lip@AB were first evaluated. SOD could convert ·O_2_^−^ into O_2_ and hydrogen peroxide (H_2_O_2_), playing a critical role in maintaining the cellular redox balance. The SOD‐like activity was assessed using nitro blue tetrazolium chloride (NBT), which reacts with ·O_2_^−^ to form brown formazan with a characteristic absorption at 560 nm. AB alone exhibited negligible ·O_2_^−^ inhibition capability (less than 10% elimination), and turned dark brown upon light exposure. However, HPB, HPB@Lip, and HPB@Lip@AB all exhibited potent ·O_2_^−^ scavenging activity in a concentration‐dependent manner. At an HPB@Lip@AB concentration of 4 mg/mL, the ·O_2_^−^ inhibition rates reached 96.3%, compared to 93.1% and 73.1% for HPB and HPB@Lip at the corresponding concentrations, respectively (Figure 3D and Figure S6A). Although SOD eliminates the highly toxic ·O_2_^−^, the resulting product H_2_O_2_ remains toxic and can cause oxidative damage. CAT, another key enzyme in the antioxidant defense system, could further decompose H_2_O_2_ into harmless H_2_O and O_2_. The CAT‐like activity was evaluated by monitoring the decomposition of H_2_O_2_. Upon mixing with H_2_O_2_, HPB, HPB@Lip, and HPB@Lip@AB all generated O_2_ bubbles, whereas no bubbles were observed in the AB alone group. At an HPB@Lip@AB concentration of 4 mg/mL, the dissolved O_2_ increased to 22.7 mg/L, whereas HPB and HPB@Lip generated 16.3 and 18.1 mg/L, respectively (Figure 3E and Figure S6B). POD, another crucial enzyme in maintaining the oxidant‐antioxidant balance, can also catalyze H_2_O_2_ into ·OH. The POD‐like activity was evaluated by tetramethylbenzidine (TMB), which could be oxidized into blue oxTMB with a characteristic absorption at 650 nm. As the concentrations of HPB, HPB@Lip, and HPB@Lip@AB increased, the color of TMB gradually turned blue and even green, accompanied by a progressive increase in Δabsorbance at 650 nm. In contrast, the AB group remained colorless with no change in absorbance (Figure 3F and Figure S6C).

Collectively, these results indicate that HPB@Lip@AB retains multienzyme‐like activities that are primarily associated with HPB, while AB appears to make a minimal contribution under the tested conditions. Liposome coating showed no substantial effect on the measured enzymatic performance.

Apart from multienzyme‐like activity, the antioxidant capability of HPB@Lip@AB was also evaluated. ·OH is the most reactive of ROS and can react with various molecules to cause cellular damage. The ·OH scavenging capability was evaluated using salicylic acid as an indicator, which can be oxidized by ·OH to purple 2,3‐dihydroxybenzoic acid. HPB, HPB@Lip, AB and HPB@Lip@AB all exhibited strong ·OH scavenging capabilities in a concentration‐dependent manner, with the purple color noticeably lightened. At least 70% of ·OH was scavenged by 4 mg/mL HPB@Lip@AB (or the corresponding amounts of HPB and AB) (Figure 3G and Figure S6D). This indicates that both HPB and AB contribute substantially to the ·OH scavenging capability of HPB@Lip@AB. In addition, this effect appeared to be somewhat attenuated by the liposome coating. Apart from ROS, the reactive nitrogen species (RNS) scavenging capabilities were further evaluated using diphenylpicrylhydrazyl (DPPH) and 2,2'‐azino‐bis (3‐ethylbenzothiazoline‐6‐sulfonic acid) diammonium salt (ABTS) reagents. Both HPB and HPB@Lip showed limited ABTS·^+^ scavenging capacities (less than 40%), whereas AB and HPB@Lip@AB exhibited robust scavenging activity (approximately 100%) (Figure 3H and Figure S6E). This was because highly antioxidant H_2_ was produced from AB. Furthermore, HPB, HPB@Lip, AB, and HPB@Lip@AB all showed moderate DPPH· scavenging capacities (Figure 3I and Figure S6F). Taken together, these results demonstrate that HPB@Lip@AB can effectively scavenge ·O_2_^−^, H_2_O_2_, ·OH, and RNS through the multi‐enzyme‐like activities of HPB and the antioxidant activity of H_2_ (Figure 3J).

### 2.4 Nanomotor Motion Behavior

The tear film barrier and the short retention time are the key factors that limit the efficacy of drugs in the eyes. Nanomotor‐based drug delivery systems could effectively overcome these limitations. Various studies have proven that high levels of ROS (including H_2_O_2_) exist in the tear film of DED patients [44]. Leveraging this environment, the CAT‐like activity of HPB@Lip@AB nanomotors might catalyze H_2_O_2_ to generate O_2_ bubbles, producing propulsion to overcome the tear film barrier. To investigate this, we examined the motion behavior of C6‐labeled HPB@Lip@AB nanomotors in STF containing H_2_O_2_. Although the exact H_2_O_2_ concentration in the tear film of DED patients has not been definitively reported, we selected 1 and 10 mm H_2_O_2_ to simulate oxidative stress conditions of varying severity based on concentrations commonly used in both DED oxidative stress models and nanomotor propulsion studies. The movements of HPB@Lip and HPB@Lip@AB were recorded under an inverted fluorescence microscope with a charge‐coupled device (CCD) camera, and the trajectories were analyzed using the Tracker software. Within 20 s, both HPB@Lip and HPB@Lip@AB exhibited inconspicuous Brownian motion in STF, characterized by random, constrained displacement. In contrast, upon exposure to 1 and 10 mm H_2_O_2_, their motion was markedly enhanced in a concentration‐dependent manner, with greater displacement observed at higher H_2_O_2_ concentrations (Figure 3K and Movies S1–S6). To further quantify the nanomotor trajectories, critical parameters including the mean squared displacement (MSD), effective diffusion coefficient (D_eff_), and average speed (*v*) were calculated within 4 s. MSD was calculated from Equation 7, and the plots of MSD versus the time interval (Δ*t*) were shown in Figure 3L. MSD increased in a time‐dependent manner and remained comparable between HPB@Lip and HPB@Lip@AB under all tested conditions. At Δt = 4 s, the MSD values in STF were 3.9 and 12.2 µm^2^, respectively, which increased to 61.6 and 66.9 µm^2^ at 1 mM H_2_O_2_, and further to 250.9 and 296.9 µm^2^ at 10 mM H_2_O_2_. At this time point, the D_eff_ values of HPB@Lip and HPB@Lip@AB were below 1 µm^2^/s, increased to approximately 4 µm^2^/s at 1 mm H_2_O_2_ and 18 µm^2^/s at 10 mm H_2_O_2_ (Figure 3M). Consistently, the values of v also increased with H_2_O_2_ concentration, confirming that H_2_O_2_ acts as an effective fuel to enhance the propulsion of both HPB@Lip and HPB@Lip@AB nanomotors (Figure 3N).

Taking the results of in vitro H_2_ release, multienzyme‐like activities, and nanomotor motion behavior together, we could infer the process and advantages of HPB@Lip@AB for treating DED. As seen in Figure 3O, HPB@Lip@AB nanomotors could quickly pass through the tear film barrier, propelled by O_2_ bubbles generated from H_2_O_2_ decomposition. They were then effectively taken up by the corneal epithelial cells, where they utilized the SOD‐like, CAT‐like, and POD‐like activities to scavenge ROS. Furthermore, after cellular internalization, AB may be released from HPB@Lip@AB and generate H_2_ in acidic intracellular environments, which also serves as a powerful antioxidant to scavenge ROS.

### 2.5 HPB@Lip@AB Targets Mitochondrial Dysfunction to Alleviate Cellular Apoptosis and Inflammation In Vitro

**FIGURE 4:**
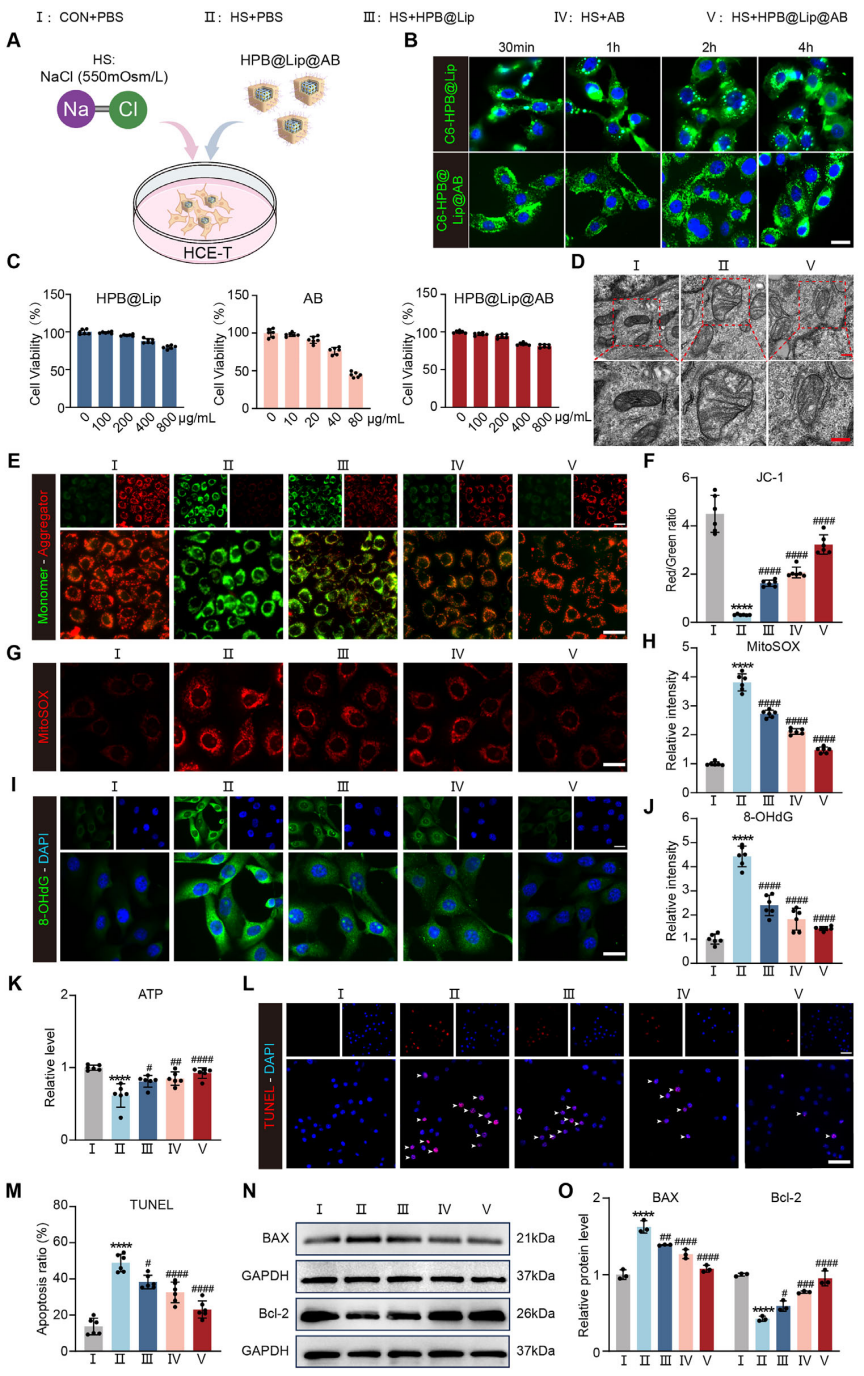
In vitro antioxidant and anti‐apoptotic effects of HPB@Lip@AB. (A) Schematic diagram of the in vitro experiment of HPB@Lip@AB. (B) Cellular uptake of C6‐labeled HPB@Lip and HPB@Lip@AB in HCE‐T cells, Scale bar, 25 µm (n = 3). (C) Viability of HCE‐T cells following 24 h treatment with different concentrations of HPB@Lip, AB, or HPB@Lip@AB (n = 6). (D) Representative TEM images of the mitochondrial ultrastructure in HCE‐T cells. The next row is an enlarged illustration of the red boxes of the respective images in the previous row, Scale bar, 2 µm (n = 3). (E, F) ΔΨm of HCE‐T cells analyzed by JC‐1 staining, accompanied by statistical data, Scale bar, 100 µm (n = 6; one‐way ANOVA with Tukey's multiple comparisons test). (G, H) Representative MitoSOX fluorescence images of HCE‐T cells, accompanied by statistical data, Scale bar, 25 µm (n = 6; one‐way ANOVA with Tukey's multiple comparisons test). (I, J) Representative immunofluorescence images of 8‐OHdG staining in HCE‐T cells, accompanied by statistical data, Scale bar, 25 µm (n = 6; one‐way ANOVA with Tukey's multiple comparisons test). (K) Relative ATP levels of HCE‐T cells. (n = 6; one‐way ANOVA with Tukey's multiple comparisons test). (L, M) Representative TUNEL staining images of HCE‐T cells, accompanied by statistical data. White arrows indicate apoptotic cells, Scale bar, 200 µm (n = 6; one‐way ANOVA with Tukey's multiple comparisons test). (N) Protein expression levels of BAX and Bcl‐2 in HCE‐T cells were determined by Western blotting; GAPDH was used as the loading control. (O) Quantitative statistics for the protein levels of BAX and Bcl‐2. (n = 3; one‐way ANOVA with Tukey's multiple comparisons test). Data are presented as mean ± SD; ^****^ *p* < 0.0001 vs. CON + PBS group; ^#^ *p* < 0.05, ^##^ *p* < 0.01, ^###^ *p* < 0.001, and ^####^ *p* < 0.0001 vs. HS + PBS group.

Tear hyperosmolarity is a hallmark feature of DED. Here, an in vitro hyperosmotic stress (HS) model was established by exposing human corneal epithelial (HCE‐T) cells to a hyperosmotic medium (550 mOsm/L), after which the cells were treated with HPB@Lip@AB to explore its protective effect at the cellular level (Figure 4A). To evaluate the cellular internalization capacity, C6‐labeled HPB@Lip and HPB@Lip@AB were incubated with HS‐treated HCE‐T cells. As shown in Figure 4B and Figure S7, bright green fluorescence was observed in the cytoplasm of cells, and the intensity increased over time. This indicated that HPB@Lip@AB could be easily internalized by corneal epithelial cells, which facilitated subsequent intracellular ROS scavenging. Next, to exclude the potential interference of nanomaterial toxicity on the evaluation of their protective efficacy, we incubated HCE‐T cells with varying concentrations of HPB@Lip, AB, and HPB@Lip@AB and assessed cell viability using the 3‐(4,5‐dimethylthiazol‐2‐yl)‐2,5‐diphenyltetrazolium bromide (MTT) assay (Figure 4C). The results demonstrated that cell viability remained above 90% at AB concentrations below 20 µg/mL, indicating that concentrations below 20 µg/mL can be considered safe. In contrast, both HPB@Lip and HPB@Lip@AB showed minimal cytotoxicity, maintaining cell viability above 85% even at concentrations up to 400 µg/mL.

To examine the mitochondrial protective role of HPB@Lip@AB, we assessed its capacity to preserve mitochondrial integrity. TEM analysis revealed the damaging effect of HS on the mitochondrial ultrastructure of HCE‐T cells. Mitochondria in the normal control group maintained a typical ellipsoidal shape with dense and uniformly distributed cristae. In contrast, the HS group showed significant pathological changes: mitochondria appeared swollen, deformed, and spherical, with extensive fragmentation of cristae and matrix vacuolization. This mitochondrial structural damage was significantly reversed in the HPB@Lip@AB treatment group, where mitochondrial morphology was largely restored, cristae were reorganized into continuous lamellar structures, and matrix vacuolization was significantly reduced (Figure 4D). ΔΨm is a fundamental indicator of mitochondrial function. We performed JC‐1 staining to evaluate the protective effect of HPB@Lip@AB on ΔΨm in HS‐treated HCE‐T cells (Figure 4E,F). In healthy mitochondria, JC‐1 forms J‐aggregates emitting red fluorescence; however, mitochondrial dysfunction triggers depolarization, causing JC‐1 dissociation into monomers that emit green fluorescence. The red/green fluorescence ratio was used to evaluate ΔΨm changes. Compared with the control group, the HS group exhibited significant mitochondrial depolarization, characterized by a diffuse increase in green fluorescence and a significant decrease in red fluorescence, with the red/green ratio decreasing to 0.31 ± 0.02. Treatment with either HPB@Lip or AB partially reversed this depolarization, with the red/green ratios recovering to 1.62 ± 0.13 and 2.07 ± 0.22, respectively. Notably, the red/green ratio in the HPB@Lip@AB group was 3.23 ± 0.40, suggesting that HPB@Lip@AB exerts a protective effect by preserving ΔΨm.

To evaluate oxidative stress levels, HCE‐T cells under HS were stained with MitoSOX and dihydroethidium (DHE) to assess mtROS and total ROS, respectively. The HS group exhibited intense fluorescence, indicating significantly elevated oxidative stress. Although HPB@Lip and AB treatments partially attenuated these signals, the HPB@Lip@AB group restored fluorescence intensities to levels comparable to the control, confirming the potent dual antioxidant capacity of HPB@Lip@AB (Figure 4G,H and Figure S8A,B). qPCR analysis revealed that HS upregulated NADPH oxidase 4 (NOX4) mRNA expression. This upregulation was partially suppressed by HPB@Lip or AB treatment, with the most significant inhibition observed in the HPB@Lip@AB group (Figure S8C).

Excessive ROS accumulation impairs core mitochondrial defense mechanisms, notably depleting the key antioxidant enzyme SOD2. This collapse in antioxidant capacity disrupts redox homeostasis, leading to the accumulation of 8‐OHdG, a marker of oxidative DNA damage. Immunofluorescence staining confirmed that HS significantly decreased SOD2 expression and increased 8‐OHdG levels. Treatment with HPB@Lip@AB effectively reversed this imbalance, restoring SOD2 expression and reducing 8‐OHdG accumulation to near‐normal levels—an effect significantly superior to the partial restoration seen with HPB@Lip or AB alone (Figure S8D,E and Figure 4I,J). Furthermore, ATP measurement showed that HS impaired mitochondrial energy production. While treatment with HPB@Lip and AB already partially restored ATP levels to 81.12% ± 8.12% and 84.97% ± 9.18% of control, respectively, HPB@Lip@AB achieved a nearly complete recovery (92.51% ± 7.23% of control). These results show that HPB@Lip@AB effectively rescues mitochondrial energy metabolism and mitigates HS‐induced injury in HCE‐T cells (Figure 4K).

To evaluate the anti‐apoptotic effect of HPB@Lip@AB, we performed TUNEL staining and quantified the expression of key mitochondrial apoptotic regulators, BAX and Bcl‐2 (Figure 4L–O). HS markedly increased the percentage of TUNEL‐positive cells and induced a pro‐apoptotic shift, characterized by upregulated BAX and downregulated Bcl‐2. Treatment with HPB@Lip@AB dramatically attenuated apoptosis, significantly reducing the percentage of TUNEL‐positive cells, downregulating BAX, and restoring Bcl‐2 expression. Notably, this combined effect surpassed that of either HPB@Lip or AB administered alone, demonstrating potent combined anti‐apoptotic effects.

In addition, we performed immunofluorescence analysis of NLRP3, Caspase‐1, and IL‐1β (Figure S9A–F). The fluorescence intensities of NLRP3, Caspase‐1, and IL‐1β in the HS group were all significantly increased. In contrast, the HPB@Lip@AB group significantly inhibited this inflammatory response, reducing the fluorescence intensity of all three markers. Although the single‐agent treatment groups (HS + HPB@Lip and HS + AB) also showed partial improvement in the inflammatory state, their effects were significantly weaker than those of the HPB@Lip@AB treatment group. IL‐1β mRNA levels showed the same pattern (Figure S9G). These results indicate that HPB@Lip@AB can effectively inhibit the NLRP3 inflammasome pathway, thereby synergistically alleviating hyperosmolarity‐induced inflammation.

### 2.6 HPB@Lip@AB Suppresses Dry Eye Disease In Vivo

**FIGURE 5:**
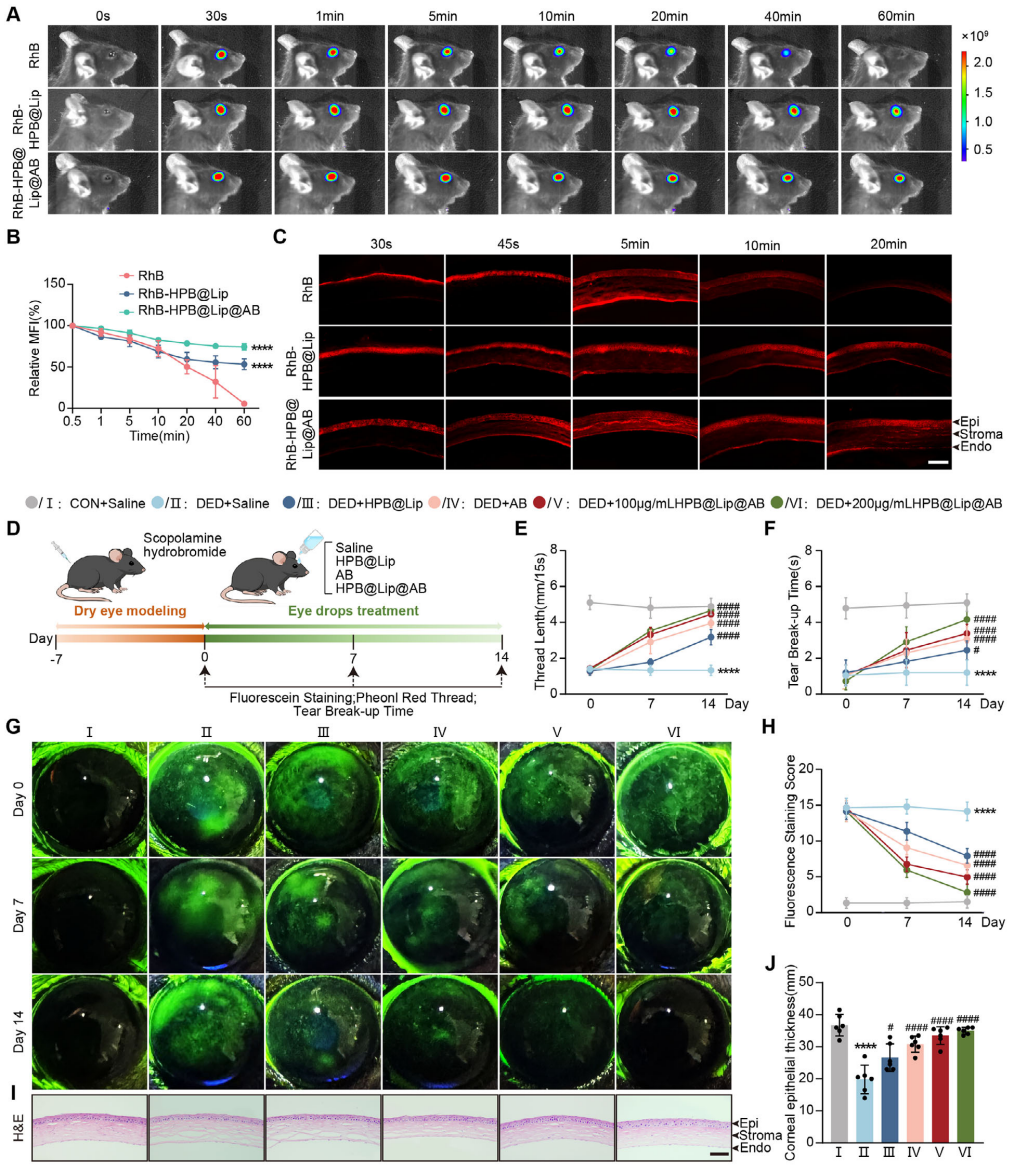
In vivo therapeutic efficacy of HPB@Lip@AB against DED. (A) Representative in vivo fluorescence images of DED mouse eyes at various time points post‐administration of RhB, RhB‐HPB@Lip, or RhB‐HPB@Lip@AB (n = 3). (B) Quantification of relative fluorescence intensity following topical administration of RhB, RhB‐HPB@Lip, and RhB‐HPB@Lip@AB (n = 3; two‐way ANOVA with Tukey's multiple comparisons test). (C) Representative fluorescence images of frozen ocular surface sections from DED mice at indicated time points after administration of RhB, RhB‐HPB@Lip, or RhB‐HPB@Lip@AB, Scale bar, 50 µm. (D) Schematic illustration of the DED model and treatment regimen. DED was induced by subcutaneous injection of scopolamine hydrobromide (1.5 mg/0.3 mL, three times daily for 7 days). From day 0, mice received bilateral topical administration (5 µL per eye) of saline, HPB@Lip, AB, or HPB@Lip@AB (100 µg/mL or 200 µg/mL). Ocular surface assessments were performed on days 0, 7, and 14. (E) Tear secretion measured by phenol red thread test (n = 6; two‐way ANOVA with Tukey's multiple comparisons test). (F) TBUT (n = 6; two‐way ANOVA with Tukey's multiple comparisons test). (G) Representative corneal fluorescein staining images. (H) Fluorescein staining scores (n = 6; two‐way ANOVA with Tukey's multiple comparisons test). (I) Representative H&E staining images of corneal tissues showing epithelial and stromal structures, Scale bar, 50 µm. (J) Quantitative analysis of corneal epithelial thickness. (n = 6; one‐way ANOVA with Tukey's multiple comparisons test). Data are presented as mean ± SD; ^****^ *p* < 0.0001 vs. RhB group (A, B) or CON + Saline group (E‐J); ^#^ *p* < 0.05 and ^####^ *p* < 0.0001 vs. DED + Saline group. Epi, corneal epithelium; Stroma, corneal stroma; Endo, corneal endothelium.

Ocular surface retention and corneal permeability are key factors determining the therapeutic effect of drugs in the eye. However, the existence of ocular surface barriers inevitably affects the ocular delivery efficiency of drugs or nanoparticles. To evaluate the ability of HPB@Lip@AB nanomotors to overcome ocular surface barriers, we labeled them with Rhodamine B (RhB) and tracked precorneal retention by real‐time in vivo fluorescence imaging. As seen in Figure 5A, strong fluorescence signals were observed in the mice treated with free RhB, RhB‐HPB@Lip, and RhB‐HPB@Lip@AB during the first 30 s post‐administration. Subsequently, the signal in the free RhB group decayed rapidly and disappeared at 60 min. However, the decline in fluorescence signals in the RhB‐HPB@Lip and RhB‐HPB@Lip@AB groups was significantly delayed. Quantitative analysis at 60 min confirmed superior retention for RhB‐HPB@Lip@AB, with its fluorescence intensity being 13.1‐fold and 1.4‐fold higher than that of the free RhB and RhB‐HPB@Lip groups, respectively (Figure 5B). Furthermore, the corneal penetration of HPB@Lip@AB nanomotors was also evaluated using ex vivo corneal cryosections obtained from DED mice. Following topical administration of RhB, RhB‐HPB@Lip, and RhB‐HPB@Lip@AB, corneal tissues were harvested at designated intervals and cryosectioned for systematic evaluation of fluorescence distribution across corneal layers. The results showed that RhB‐HPB@Lip and RhB‐HPB@Lip@AB penetrated the entire corneal epithelium within 30 s and reached the full corneal thickness by 45 s, whereas free RhB remained confined to the epithelial layer. Regarding retention, RhB‐HPB@Lip and RhB‐HPB@Lip@AB maintained strong fluorescence signals from 5 to 20 min after administration, while the signal from free RhB significantly declined after 10 min. These findings demonstrate that the nanomotor motion of HPB@Lip and HPB@Lip@AB enables rapid penetration and prolonged retention on the ocular surface (Figure 5C).

To evaluate the therapeutic potential of HPB@Lip@AB for DED, a murine model was established via subcutaneous injections of scopolamine hydrobromide (1.5 mg/0.3 mL, three times daily) for 7 days, as confirmed by the presence of corneal surface defects and reduced tear secretion. DED mice were then treated topically (5 µL per eye, twice daily for 14 days) with saline, HPB@Lip, AB, or HPB@Lip@AB at concentrations of 100 and 200 µg/mL (Figure 5D). The phenol red thread test showed that tear secretion was significantly reduced in DED mice (1.37 ± 0.30 mm/15 s) compared with controls (5.17 ± 0.48 mm/15 s). Treatment with HPB@Lip (3.33 ± 0.45 mm/15 s) or AB (4.17 ± 0.26 mm/15 s) partially restored secretion, whereas HPB@Lip@AB at 100 µg/mL (4.70 ± 0.14 mm/15 s) and 200 µg/mL (4.92 ± 0.37 mm/15 s) partially restored tear secretion (Figure 5E). Tear film breakup time (TBUT), a measure of tear film stability, averaged 5.33 ± 0.51 s in controls and was markedly reduced to 1.17 ± 0.75 s in DED mice. All treatments improved TBUT, with the 200 µg/mL HPB@Lip@AB group showing the greatest recovery (4.33 ± 0.82 s), highlighting its therapeutic advantage (Figure 5F). Corneal epithelial damage, assessed by fluorescein sodium staining (16‐point scale), was intense and persistent in DED mice (score > 10). HPB@Lip and AB treatments partially reduced staining (score > 5), but 14‐day treatment with HPB@Lip@AB, especially at 200 µg/mL, markedly weakened corneal fluorescence (Figure 5G,H).

Moreover, we evaluated changes in corneal tissue structure via hematoxylin‐eosin (H&E) staining (Figure 5I). Controls displayed tightly packed, orderly corneal epithelial layers, whereas DED mice exhibited significant epithelial thinning, superficial structural disruption, and a thickened and loosened corneal stroma. After treatment, the corneal histomorphology in each group was improved. Quantitative analysis of corneal epithelial thickness further confirmed this result: corneal epithelial thickness increased from 19.83 ± 4.44 µm in the DED group to 35.00 ± 1.05 µm in the 200 µg/mL HPB@Lip@AB group. This thickness was comparable to that of the control group (36.75 ± 3.36 µm), and significantly greater than that of the HPB@Lip group (26.67 ± 4.29 µm), the AB group (30.83 ± 2.52 µm), and the 100 µg/mL HPB@Lip@AB group (33.50 ± 2.78 µm) (Figure 5J). Moreover, compared with 0.05% CsA, 200 µg/mL HPB@Lip@AB showed significantly greater improvements in corneal fluorescein staining and corneal epithelial thickness, while showing comparable effects on tear secretion and TBUT (Figure S10A–G).

To further evaluate the therapeutic potential of HPB@Lip@AB across different DED subtypes, a benzalkonium chloride (BAC)‐induced evaporative DED model was established. HPB@Lip@AB treatment significantly improved DED‐associated ocular surface impairment and corneal structural alterations, as evidenced by enhanced tear secretion and tear film stability, reduced corneal epithelial defects, and improved corneal epithelial thickness and stromal morphology (Figure S11A–G). These results demonstrate the therapeutic efficacy of HPB@Lip@AB in both aqueous‐deficient and evaporative DED models.

### 2.7 HPB@Lip@AB Attenuates Oxidative Stress, Apoptosis and Inflammation In Vivo

**FIGURE 6:**
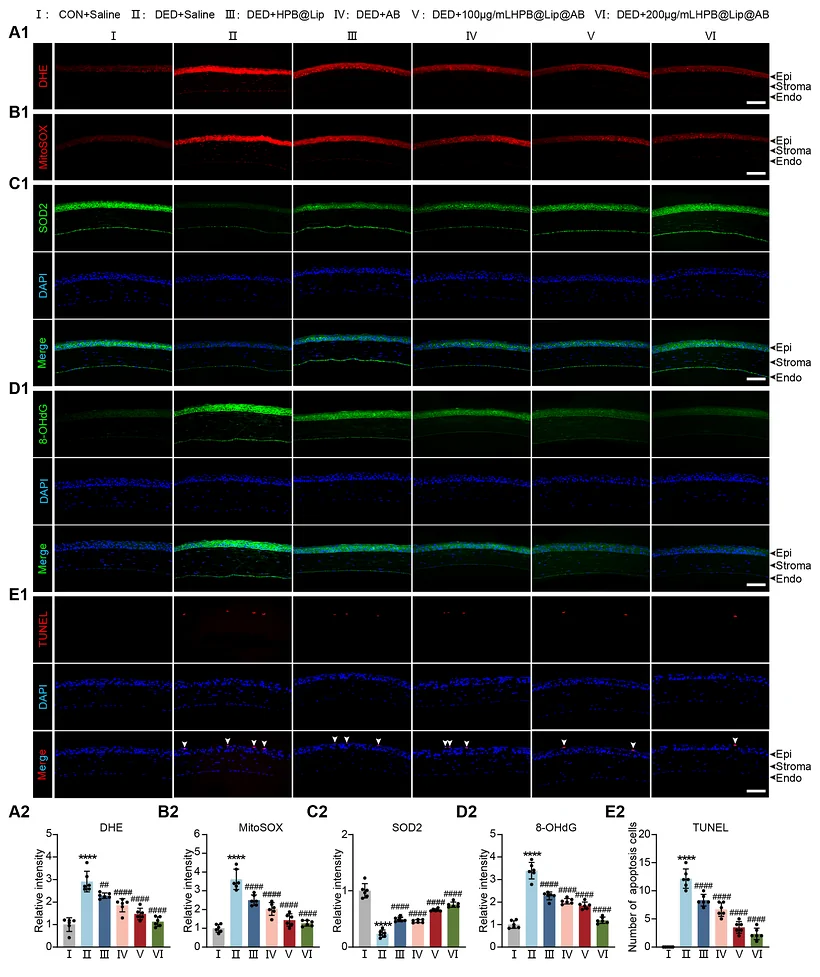
In vivo antioxidant and anti‐apoptotic effects of HPB@Lip@AB. (A) Representative DHE fluorescence images of corneal tissues, accompanied by statistical data, Scale bar, 50 µm (n = 6; one‐way ANOVA with Tukey's multiple comparisons test). (B) Representative MitoSOX fluorescence images of corneal tissues, accompanied by statistical data, Scale bar, 50 µm (n = 6; one‐way ANOVA with Tukey's multiple comparisons test). (C) Representative immunofluorescence images of corneal tissues stained with SOD2, accompanied by statistical data, Scale bar, 50 µm (n = 6; one‐way ANOVA with Tukey's multiple comparisons test). (D) Representative immunofluorescence images of corneal tissues stained with 8‐OHdG, accompanied by statistical data, Scale bar, 50 µm (n = 6; one‐way ANOVA with Tukey's multiple comparisons test). (E) Representative TUNEL staining images of the corneal tissues, accompanied by statistical data. White arrows indicate apoptotic cells, Scale bar, 50 µm (n = 6; one‐way ANOVA with Tukey's multiple comparisons test). Data are presented as mean ± SD; ^****^ *p* < 0.0001 vs. CON + Saline group; ^##^ *p* < 0.01, and ^####^ *p* < 0.0001 vs. DED + Saline group. Epi, corneal epithelium; Stroma, corneal stroma; Endo, corneal endothelium.

To further elucidate the mechanisms underlying the therapeutic effects of HPB@Lip@AB on DED, we assessed key markers of oxidative stress, inflammation, and apoptosis in corneal tissues. This included evaluation of ROS, SOD2, 8‐OHdG, TUNEL staining, and inflammasome components (NLRP3, Caspase‐1, and IL‐1β). Oxidative stress in the corneal epithelium was specifically probed using DHE and MitoSOX staining (Figure 6A,B). As expected, only weak fluorescence was detected in the control group, while the DED group showed intense DHE and MitoSOX fluorescence, indicating substantial accumulation of ROS. The fluorescence intensity was reduced after treatment with HPB@Lip or AB, confirming their partial antioxidant effects. Notably, HPB@Lip@AB demonstrated a dose‐dependent protective effect: the 200 µg/mL group showed fluorescence signals close to normal levels, which was superior to the 100 µg/mL group, thereby verifying its dose‐dependent antioxidant effect.

Additionally, we assessed the expression levels of SOD2 and 8‐OHdG via immunofluorescence staining (Figure 6C,D). Compared to normal controls, the DED group showed significantly reduced SOD2 fluorescence intensity and elevated 8‐OHdG fluorescence intensity. HPB@Lip@AB treatment reversed these changes in a dose‐dependent manner. The 200 µg/mL group simultaneously restored SOD2 and significantly reduced the level of 8‐OHdG, outperforming HPB@Lip, AB, and 100 µg/mL HPB@Lip@AB.

Moreover, we evaluated corneal epithelial cell apoptosis using TUNEL staining. As shown in Figure 6E, the control corneal epithelium contained almost no TUNEL‐positive cells, whereas the DED group exhibited TUNEL‐positive signals, indicating DED‐induced corneal epithelial cell apoptosis. Quantitative analysis of apoptotic cell numbers revealed that both HPB@Lip and AB treatments significantly reduced apoptosis compared to the DED group. HPB@Lip@AB exerted an even more potent anti‐apoptotic effect, with a significant further reduction at both 100 and 200 µg/mL. The effect of the 200 µg/mL dose was superior to that of the 100 µg/mL dose, indicating a dose‐dependent therapeutic effect.

Furthermore, we detected the expression levels of NLRP3, Caspase‐1, and IL‐1β in key components of the inflammasome pathway (Figure S12A–C). The DED group showed significantly increased fluorescence intensities of NLRP3, Caspase‐1, and IL‐1β, indicating excessive activation of the inflammasome. Compared to the DED group, both the HPB@Lip and AB groups downregulated the expression of these markers. HPB@Lip@AB treatment exerted a dose‐dependent inhibitory effect, with the 200 µg/mL dose demonstrating superior efficacy over the 100 µg/mL dose. This improvement highlights the excellent anti‐inflammatory efficacy of HPB@Lip@AB in DED.

### 2.8 Ocular Biosafety and 30‐Day Ocular Tolerance Evaluation of HPB@Lip@AB

To further evaluate the biosafety of the complete HPB@Lip@AB formulation, healthy mice were topically administered with PBS, HPB@Lip, AB, or HPB@Lip@AB at the therapeutic dose twice daily for 14 consecutive days (Figure S13A). No significant differences in retinal thickness were observed among the treatment groups compared with the PBS control group (Figure S13B). Histological examination revealed preserved conjunctival and retinal structures without evident inflammatory infiltration, tissue degeneration, or photoreceptor layer abnormalities. In addition, H&E staining of major organs, including the heart, liver, spleen, lung, and kidney, showed no detectable pathological alterations, indicating favorable systemic biosafety of HPB@Lip@AB (Figure S13C).

Ocular tolerance was subsequently evaluated after 30 consecutive days of continuous topical administration of HPB@Lip@AB at the therapeutic dose (Figure S13D). Intraocular pressure (IOP) remained stable throughout the administration period, indicating that HPB@Lip@AB had no detectable impact on IOP under the tested conditions (Figure S13E). Furthermore, no structural abnormalities were observed in ocular tissues, including the cornea, conjunctiva, and retina, after 30 days of administration (Figure S13F). In addition, retinal thickness analysis revealed no significant differences among the groups, indicating the absence of detectable retinal structural alterations following HPB@Lip@AB treatment (Figure S13G).

Collectively, these findings demonstrate that HPB@Lip@AB exhibits favorable ocular and systemic biosafety as well as excellent ocular tolerability over the 30‐day study period under the tested conditions, supporting its potential for safe ophthalmic application.

### 2.9 ALDH1L2 Mediates H_2_’s Therapeutic Effects in DED

**FIGURE 7:**
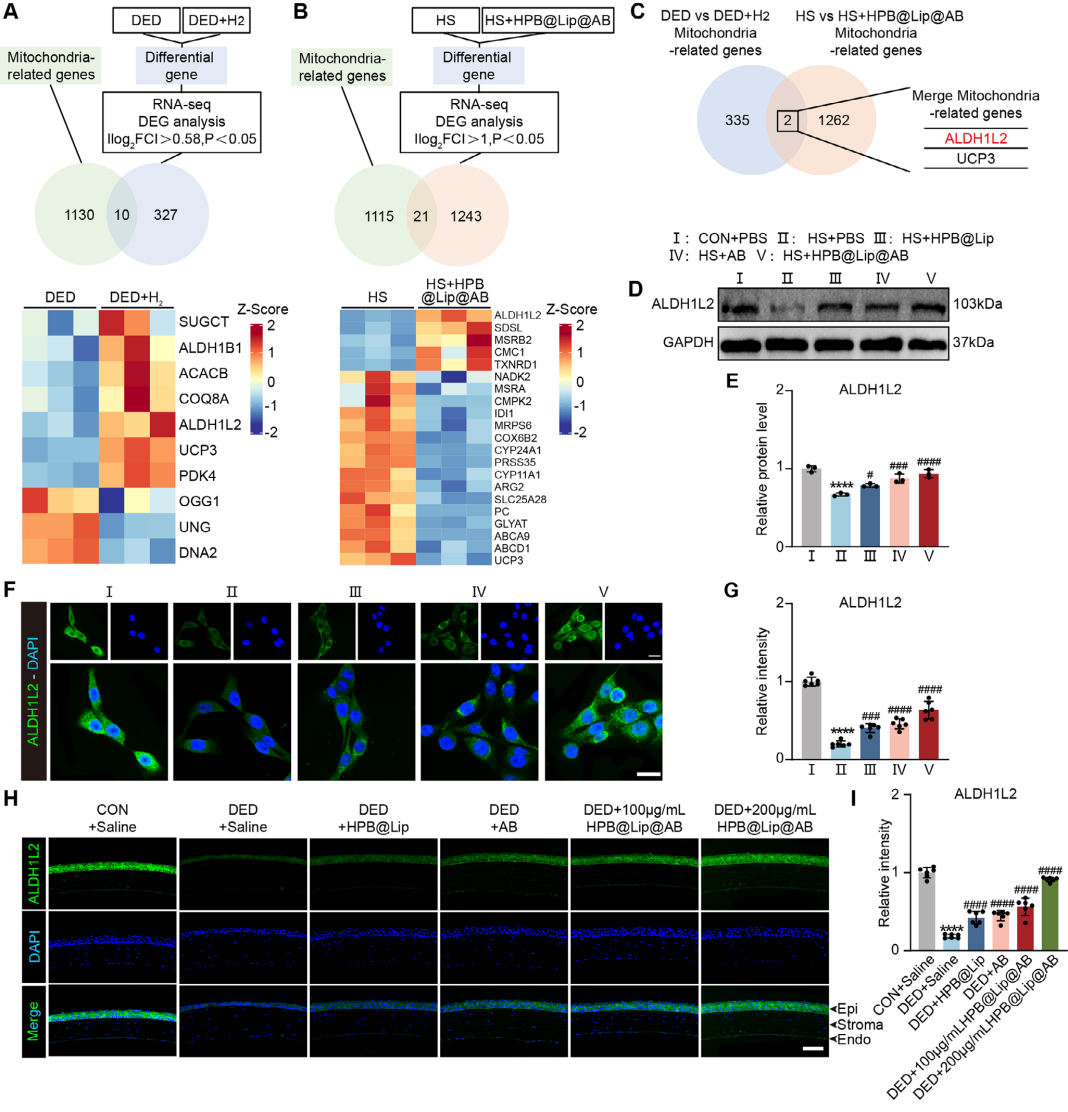
Identification of differentially expressed mitochondria‐related genes. (A) Transcriptome analysis of the mouse corneal tissues from DED and DED + H_2_ groups by bulk RNA‐seq (n = 3 per group). Mitochondria‐related DEGs were identified as the intersection between DEGs (|log_2_FC| > 0.58, *p* < 0.05) comparing DED vs. DED + H_2_ and a mitochondria‐related gene database. The heatmap shows expression levels of the 10 identified mitochondria‐related DEGs. (B) Transcriptome analysis of HCE‐T cells from HS and HS + HPB@Lip@AB groups by bulk RNA‐seq (n = 3 per group). Mitochondria‐related DEGs were identified as the intersection between DEGs (|log_2_FC| > 1, *p* < 0.05) comparing HS vs. HS + HPB@Lip@AB and a mitochondria‐related gene database. The heatmap shows expression levels of the 21 identified mitochondria‐related DEGs. (C) Venn diagram analysis revealed two shared mitochondria‐related DEGs, ALDH1L2 and UCP3 (uncoupling protein 3), between corneal tissues (DED vs. DED + H_2_) and HCE‐T cells (HS vs. HS + HPB@Lip@AB) transcriptomic profiles. (D) Protein levels of ALDH1L2 in HCE‐T cells were determined by Western blotting; GAPDH was used as the loading control. (E) Quantitative analysis of ALDH1L2 protein expression (n = 3; one‐way ANOVA with Tukey's multiple comparisons test). (F, G) Representative immunofluorescence images of HCE‐T cells stained with ALDH1L2, accompanied by statistical data, Scale bar, 25 µm (n = 6; one‐way ANOVA with Tukey's multiple comparisons test). (H, I) Representative immunofluorescence images of corneal tissues stained with ALDH1L2, accompanied by statistical data, Scale bar, 50 µm (n = 6; one‐way ANOVA with Tukey's multiple comparisons test). Data are presented as mean ± SD; ^****^ *p* < 0.0001 vs. CON + PBS group (D‐G) or CON + Saline group (H‐I); ^#^ *p* < 0.05, ^###^ *p* < 0.001, and ^####^ *p* < 0.0001 vs. HS + PBS group (D–G) or DED + Saline group (H‐I). Epi, corneal epithelium; Stroma, corneal stroma; Endo, corneal endothelium.

To clarify the key molecular targets underlying HPB@Lip@AB‐mediated amelioration of DED via mitochondrial function, we performed transcriptome sequencing analysis. First, DED mice were established and administered either saline or H_2_‐rich saline via eye drops (5 times daily for 14 days). Corneal tissues were harvested for RNA sequencing. Comparative analysis between DED and DED + H_2_ groups (n = 3 per group) identified 337 differentially expressed genes (DEGs) (|log_2_FC| > 0.58, *p* < 0.05). These DEGs were subsequently intersected with the MitoCarta 3.0 mitochondrial gene database (1140 curated genes), yielding 10 mitochondria‐associated DEGs (Figure 7A). Meanwhile, we performed transcriptome sequencing analysis on HCE‐T cells treated with HS and HS + HPB@Lip@AB (n = 3 per group). In the HS‐treated HCE‐T cells, a total of 1285 DEGs were identified (|log_2_FC| > 1 and *p* < 0.05). Further intersection analysis of these DEGs with the MitoCarta 3.0 mitochondrial gene database (1136 curated genes) ultimately yielded 21 mitochondria‐associated DEGs (Figure 7B). Notably, ALDH1L2 was significantly upregulated following H_2_ treatment in both DED mouse corneal tissues and HS‐induced HCE‐T cells (Figure 7C). Western blot analysis of HCE‐T cells confirmed that the downregulation of ALDH1L2 under HS was effectively reversed by HPB@Lip@AB treatment (Figure 7D,E). Consistently, immunofluorescence staining in both corneal tissues and HCE‐T cells showed that ALDH1L2 expression, which was markedly reduced under HS, was significantly restored following HPB@Lip@AB administration (Figure 7F–I). Collectively, these findings indicate that ALDH1L2 likely serves as a key molecular mediator through which H_2_ exerts its therapeutic effects in DED.

**FIGURE 8:**
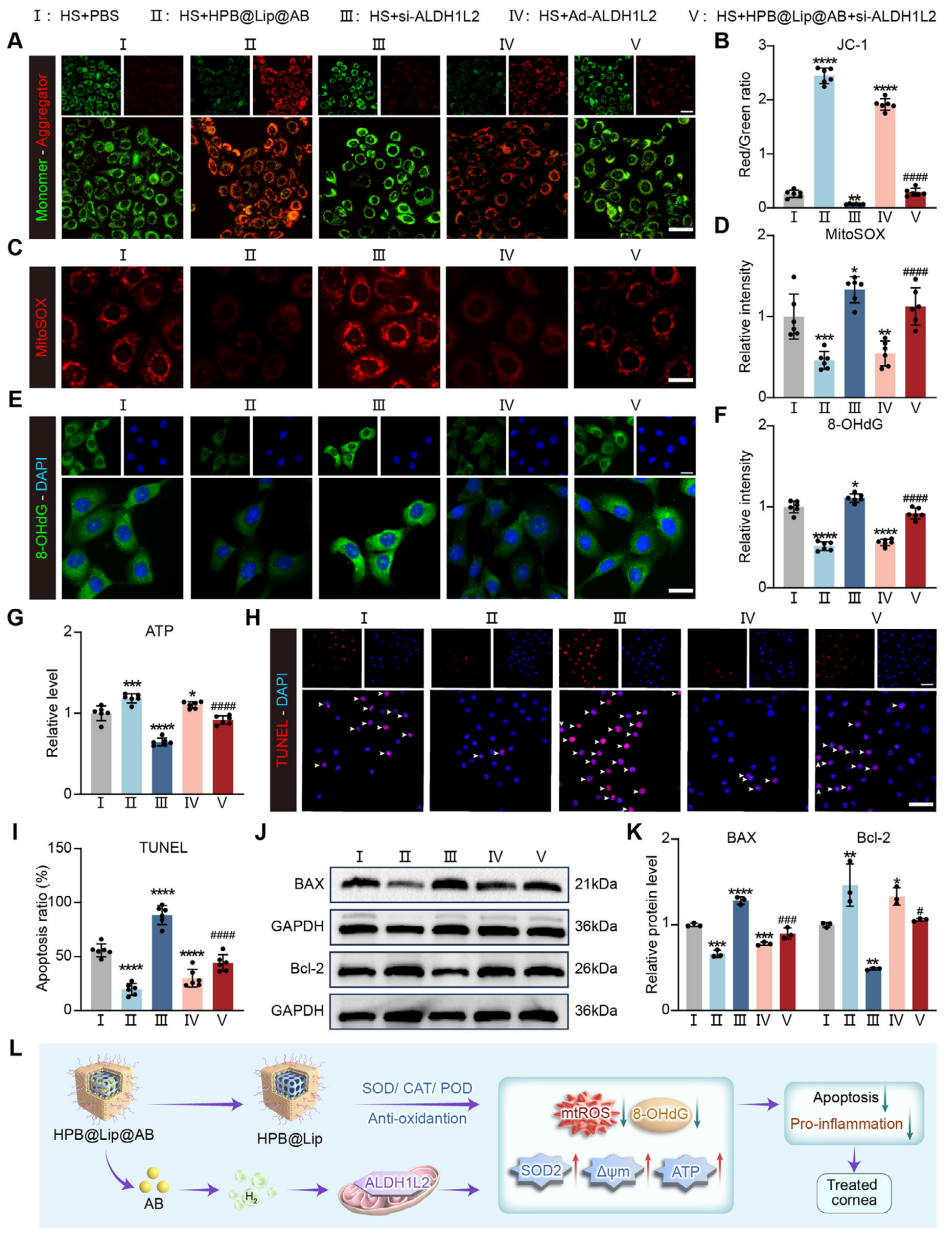
In vitro H_2_ inhibits oxidative stress and cell apoptosis by regulating ALDH1L2. (A, B) ΔΨm of HCE‐T cells analyzed by JC‐1 staining, accompanied by statistical data, Scale bar, 100 µm (n = 6; one‐way ANOVA with Tukey's multiple comparisons test). (C, D) Representative MitoSOX fluorescence images of HCE‐T cells, accompanied by statistical data, Scale bar, 25 µm (n = 6; one‐way ANOVA with Tukey's multiple comparisons test). (E, F) Representative immunofluorescence images of HCE‐T cells stained with 8‐OHdG, accompanied by statistical data, Scale bar, 25 µm (n = 6; one‐way ANOVA with Tukey's multiple comparisons test). (G) Relative ATP levels of HCE‐T cells (n = 6; one‐way ANOVA with Tukey's multiple comparisons test). (H, I) Representative TUNEL staining images of HCE‐T cells, accompanied by statistical data. White arrows indicate apoptotic cells, Scale bar, 200 µm (n = 6; one‐way ANOVA with Tukey's multiple comparisons test). (J) Protein expression levels of BAX and Bcl‐2 in HCE‐T cells were determined by Western blotting; GAPDH was used as the loading control. (K) Quantitative statistics for the protein levels of BAX and Bcl‐2 (n = 3; one‐way ANOVA with Tukey's multiple comparisons test). (L) Schematic diagram of HPB@Lip@AB alleviating cellular apoptosis and inflammation. Data are presented as mean ± SD; ^*^ *p* < 0.05, ^**^ *p* < 0.01, ^***^ *p* < 0.001, and ^****^ *p* < 0.0001 vs. HS + PBS group; ^#^ *p* < 0.05, ^###^ *p* < 0.001, and ^####^ *p* < 0.0001 vs. HS + HPB@Lip@AB group.

To determine whether HPB@Lip@AB rescues mitochondrial dysfunction through ALDH1L2 under HS, we performed adenovirus‐mediated ALDH1L2 overexpression (Ad‐ALDH1L2) and siRNA‐based ALDH1L2 knockdown in HCE‐T cells. In HS‐treated cells, ALDH1L2 knockdown exacerbated mitochondrial damage, as evidenced by increased ΔΨm loss, increased mtROS/ROS levels and NOX4 expression, heightened 8‐OHdG accumulation, reduced SOD2 expression, and further depleted ATP levels. Conversely, ALDH1L2 overexpression effectively attenuated these HS‐induced pathologies. Critically, ALDH1L2 knockdown abolished the protective effects of HPB@Lip@AB against HS‐induced mitochondrial dysfunction. Together, these results demonstrate that HPB@Lip@AB ameliorates DED by targeting ALDH1L2 to alleviate mitochondrial dysfunction (Figure 8A–G, and Figure S14A–E).

Consistent with its role in mitochondrial function, ALDH1L2 knockdown significantly exacerbated apoptosis compared with the HS group alone. This was evidenced by a further increase in the TUNEL‐positive rate (Figure 8H,I) and concomitant upregulation of pro‐apoptotic BAX alongside downregulation of anti‐apoptotic Bcl‐2 (Figure 8J,K). Conversely, ALDH1L2 overexpression alleviated HS‐induced apoptosis. Notably, ALDH1L2 knockdown abolished the anti‐apoptotic protection conferred by HPB@Lip@AB.

Similarly, ALDH1L2 manipulation directly influenced the inflammasome pathway: knockdown upregulated the expression of NLRP3, Caspase‐1, and IL‐1β, while overexpression downregulated them. Notably, ALDH1L2 knockdown also reversed the suppressive effect of HPB@Lip@AB on the HS‐induced expression of these inflammatory mediators (Figure S15A–G).

Taken together, these findings demonstrate that HPB@Lip@AB exerts protective effects in DED, potentially involving the enzyme‐mimetic antioxidant activity of HPB and the increased ALDH1L2 expression observed following treatment, accompanied by improved mitochondrial homeostasis and reduced apoptosis and inflammatory responses (Figure 8L).

## 3 Discussion

In the present study, we demonstrated that HPB@Lip@AB could rapidly penetrate the tear film barrier through an H_2_O_2_‐responsive gas‐driven motor effect, facilitating its penetration and entry into corneal epithelial cells. After internalization, the H_2_‐related effects of HPB@Lip@AB were associated with increased expression of the mitochondrial aldehyde dehydrogenase ALDH1L2. H_2_‐mediated mitochondrial regulation, together with the intrinsic multi‐enzyme‐like antioxidant activities of HPB, synergistically improved mitochondrial function by recovering ΔΨm, increasing SOD2 expression and ATP production, while reducing mtROS accumulation and DNA oxidative damage. These combined effects further suppressed oxidative stress‐induced apoptosis and inflammatory responses, ultimately mitigating DED progression.

DED is one of the most prevalent ocular surface diseases, significantly impairing quality of life [10, 45, 46]. Although its pathogenesis is not yet fully elucidated, DED is recognized as a multifactorial pathological process involving tear film instability and ocular surface inflammation [47, 48]. Substantial evidence indicates that mitochondrial dysfunction in ocular surface tissues may lead to impaired energy metabolism. Furthermore, excessive ROS‐triggered corneal epithelial cell pyroptosis has been reported in scopolamine hydrobromide‐induced DED mice [11, 44, 49, 50]. In a DED rabbit model, inhibition of ROS coupled with suppression of TNF‐α, IL‐1β, and MMP‐9 expression restored corneal and lacrimal gland histoarchitecture, increased conjunctival goblet cell density, and also reduced cellular apoptosis, thereby alleviating DED [51]. In HS‐induced hHCECs, inhibition of the ROS/NLRP3/IL‐1β signaling axis resulted in reduced levels of ROS and 8‐OHdG [52].

Recent advances in DED therapeutics have highlighted nanomaterial‐mediated strategies targeting oxidative stress and inflammation. Current strategies include functional nanocarriers for co‐delivery of anti‐inflammatory agents (e.g., CsA) [53, 54] and antioxidant enzymes, and nanomaterials mimicking endogenous antioxidant enzymes (e.g., SOD and CAT) for direct ROS scavenging [55, 56, 57]. However, these conventional nanocarriers are constrained by the ocular surface mucus barrier and tear clearance, resulting in issues such as low corneal penetration efficiency and short retention time. Furthermore, most studies lack in‐depth analysis of the downstream molecular targets regulated by nanomaterials. Addressing these limitations remains an important challenge. Compared with conventional nanotherapeutic approaches for DED treatment, HPB@Lip@AB provides several potential advantages. Its unique gas‐driven nanomotor effect, via CAT‐like activity that decomposes H_2_O_2_ to generate O_2_, produces propulsion in the ocular‐surface microenvironment, enabling enhanced penetration across ocular surface barriers, significantly prolonging ocular retention time and reducing the frequency of administration. Additionally, the synergistic action between HPB and H_2_ contributes to the therapeutic efficacy of HPB@Lip@AB against DED.

H_2_ therapy represents an emerging antioxidant strategy that utilizes biologically safe H_2_ to alleviate oxidative stress‐associated pathological conditions [58, 59]. Its protective effects have been found in various diseases such as myocardial injury [60], spontaneous hypertension [61], acute lung injury [62], and Parkinson's disease [63]. Numerous studies have shown that H_2_ selectively scavenges ROS, restores ΔΨm and ATP production, and reduces levels of oxidized glutathione (GSSG), thereby improving mitochondrial function and exerting anti‐inflammatory and anti‐apoptotic effects [59, 64, 65]. H_2_ therapy is also widely applied in the treatment of ophthalmic diseases. A study demonstrates that H_2_ inhalation downregulates VEGF‐A expression, ameliorating retinal edema and functional deficits in branch retinal vein occlusion [19]. Prophylactic H_2_‐rich eyedrops upregulate the expression of SOD1 and peroxisome proliferator‐activated receptor gamma coactivator 1α (PGC‐1α), and inhibit inflammation, thereby suppressing corneal alkali burns [66]. Moreover, either eye drops of H_2_‐rich saline or intraperitoneal injection can alleviate histopathological damage of DED [22]. A clinical study showed that oral intake of H_2_‐rich milk enhanced tear film stability and significantly reduced the level of 8‐OHdG in tears [67]. Current H_2_‐based eye drops face limitations including monotherapeutic efficacy, unclear mechanisms, short ocular residence time, and low aqueous solubility [68]. This study selected AB as the H_2_‐releasing precursor based on its high H_2_ storage density. It has been found that AB‐loaded mesoporous silica nanoparticles (MSNs) and melatonin/AB co‐loaded mesoporous polydopamine nanoparticles (mPDA NPs) significantly inhibit ROS and inflammation through H_2_ release [26, 69].

To elucidate the mechanism underlying the action of HPB@Lip@AB, this study performed transcriptome sequencing to analyze DEGs in corneal tissues of DED mice and HS‐induced HCE‐T cells. Subsequent intersection analysis with the mitochondrial gene database MitoCarta 3.0 ultimately identified ALDH1L2 as a candidate regulator of mitochondrial function. ALDH1L2, a mitochondrial enzyme within the aldehyde dehydrogenase (ALDH) superfamily, functions as mitochondrial 10‐formyltetrahydrofolate dehydrogenase (mtFDH) [70]. This enzyme catalyzes the irreversible oxidation of 10‐formyltetrahydrofolate to tetrahydrofolate (THF) and CO_2_, coupled with concomitant reduction of NADP^+^ to NADPH. The NADPH generated from this reaction serves as an essential cofactor for maintaining mitochondrial redox homeostasis by driving glutathione reductase (GR)‐mediated conversion of GSSG to reduced glutathione (GSH) [71, 72]. Previous studies have shown that ursodeoxycholic acid ameliorates mitochondrial dysfunction and mitigates oxidative stress by activating ALDH1L2, thereby preventing cisplatin‐induced acute kidney injury [73]. Additionally, ALDH1L2 promotes mitochondrial respiration, increases ATP production, and stabilizes liver cancer cells against ROS‐induced cell damage [74]. However, its role in DED is still unclear. In this study, by performing ALDH1L2 knockdown and overexpression in HS‐treated HCE‐T cells, we confirmed that HPB@Lip@AB effectively facilitated increased ALDH1L2 expression and improved mitochondrial parameters under HS conditions. This improvement encompasses the restoration of mitochondrial ultrastructure, increased ΔΨm, SOD2 activity, and ATP production, alongside reduced mtROS and oxidative DNA damage. Collectively, these changes inhibit both apoptosis and the NLRP3/Caspase‐1/IL‐1β signaling pathway, thereby alleviating DED progression. The identification of this mechanism provides a molecular basis for the multi‐target synergistic therapeutic effect of HPB@Lip@AB.

Although our transcriptomic analyses and validation experiments indicate that H_2_ treatment is associated with increased ALDH1L2 expression under DED‐related stress conditions, the precise molecular mechanism by which H_2_ regulates ALDH1L2 expression requires further investigation.

## 4 Conclusion

In conclusion, this study developed a self‐propelled HPB@Lip@AB nanomotor‐based eye drop system that integrates H_2_ therapy, nanozyme‐mediated antioxidant activity, and enhanced ocular surface penetration for DED treatment. Through an H_2_O_2_‐responsive oxygen bubble‐driven propulsion mechanism, HPB@Lip@AB effectively overcame ocular surface barriers and improved corneal epithelial accumulation. Following cellular uptake, HPB@Lip@AB restored mitochondrial function by recovering ΔΨm, enhancing antioxidant defense and ATP production, while reducing oxidative stress, apoptosis, and inflammation. Transcriptomic analysis and functional validation identified ALDH1L2 as a potential molecular mediator of the protective effects of HPB@Lip@AB. A limitation of this study is that intracellular H_2_ generation and its direct causal relationship with ALDH1L2 regulation were not directly determined. Future studies should investigate the upstream regulatory mechanisms of ALDH1L2 and further evaluate the translational potential of this nanomotor platform for ophthalmic applications.

## 5 Materials and Methods

### 5.1 Synthesis of HPB, HPB@Lip, and HPB@Lip@AB

HPB nanozyme was synthesized according to the previously reported method [28]. Briefly, 200 mL HCl (0.01 m) solution containing 0.66 g K_3_[Fe(CN)_6_] (01020659; Adamas‐Beta Co., Ltd., Shanghai, China) and 15 g polyvinyl pyrrolidone (PVP; 011006796; Adamas‐Beta Co., Ltd.) was heated at 80°C for 20 h. The formed PB was obtained through centrifugation (12 000 rpm, 15 min) and washing with deionized water several times. Next, the PB was dispersed in 200 mL HCl (1 m) containing 1 g PVP, and reacted in a Teflon high‐pressure reaction kettle at 140°C for 4 h. Finally, the HPB nanozyme was obtained through centrifugation and washing with deionized water several times.

HPB@Lip was prepared according to previously reported methods with some modifications [75, 76]. Briefly, a mixture of lecithin (S30871; Yuanye Co., Ltd., Shanghai, China), cholesterol (S23165; Yuanye Co., Ltd.), and DSPE‐PEG2000 (S25991; Yuanye Co., Ltd.) (at a mass ratio of 3:1:1) was dissolved in dichloromethane and dried in a rotary evaporator. Then, the lipid film was hydrated with HPB solution (with the HPB/lipid mass ratio of 1:2) and sonicated in a water bath for 30 min to form crude HPB@Lip. Subsequently, the crude HPB@Lip was extruded through a series of Millipore filters (1, 0.65, 0.45, and 0.22 µm) using a hand‐held mini‐extruder. The solution was further centrifuged and washed with deionized water to obtain HPB@Lip. Finally, 200 mg HPB@Lip and 5 g AB (BD118552; Bidepharmatech Co., Ltd., Shanghai, China) were mixed in 10 mL PBS (pH = 7.4) and stirred for 24 h. The mixture was then centrifuged (12 000 rpm, 15 min) to remove the unloaded AB and the precipitate was lyophilized with a freeze‐dryer (SCIENTZ‐10N, Ningbo Scientz Biotechnology Co., Ltd., Ningbo, China) to obtain HPB@Lip@AB.

The amount of AB in HPB@Lip@AB was measured by the hydrolysis reaction method using Co‐Fe‐B as a catalyst, which was synthesized following a previously reported protocol [77]. Briefly, 20 mg Co‐Fe‐B catalyst was placed in a three‐necked round‐bottom flask. One neck was connected to the HPB@Lip@AB solution (2 mg/mL with 20 mm NaOH), and the other was connected to a 100 cm^3^ gas burette. Then, 10 mL HPB@Lip@AB solution was rapidly dropped into the flask and gently stirred to produce H_2_ gas. After 30 min, the volume of generated H_2_ gas was measured by the water‐filled gas burette using the water displacement method. The temperature was maintained at 298 K, and the pressure was 1 atm. Finally, the amount of AB as well as the loading capacity (LC) could be calculated following Equations 1 and 2.

(1) $$n_{\mathrm{AB}}=\frac{V}{3*V_{m}}\;$$

(2) $$\mathrm{LC}(\%)=\frac{M_{\mathrm{AB}}*n_{\mathrm{AB}}}{\mathrm{The}\;\mathrm{amount}\;\mathrm{of}\;\mathrm{HPB}@\mathrm{Lip}@\mathrm{AB}}\;*100$$

where *V* is the volume of generated H_2_ gas, *V*_m_ is the molar volume of gas (24.5 L/mol), *M*_AB_ is the relative molecular weight of AB (30.865 g/mol).

### 5.2 Characterization of HPB, HPB@Lip, and HPB@Lip@AB

Measurements of DLS and zeta potential were carried out on a Zetasizer ZS90 instrument (Malvern Panalytical, Malvern, Worcestershire, UK). TEM images and elemental mapping were acquired using a JEOL 2100F transmission electron microscope (JEOL Ltd., Tokyo, Japan) equipped with energy‐dispersive x‐ray spectroscopy (EDS). UV–Vis absorption spectra were recorded on a UV‐1800 PC spectrophotometer (Mapada, Shanghai, China). FTIR spectra were obtained using an IRPrestige‐21 spectrophotometer (Shimadzu Corporation, Kyoto, Japan). XRD patterns were collected on a Rigaku SmartLab diffractometer (Rigaku Corporation, Tokyo, Japan). XPS analysis was performed using an ESCALAB 250Xi spectrometer (Thermo Fisher Scientific, Waltham, MA, USA).

To investigate the colloidal stabilities of HPB, HPB@Lip, and HPB@Lip@AB, they were respectively dispersed in deionized water, PBS (pH = 7.4), STF (containing 6.8 g/L NaCl, 2.2 g/L NaHCO_3_, 1.4 g/L KCl, 0.084 g/L CaCl_2_·H_2_O, pH = 7.4) [78], DMEM medium, and DMEM supplemented with 10% FBS (DMEM + FBS). Their particle sizes were measured by DLS at 0, 24, and 48 h.

### 5.3 In Vitro H_2_ Release

The apparatuses of measuring H_2_ release were the same as that used for AB detection. Briefly, 100 mL PBS (pH = 5.6, 6.2, 6.8, 7.2, 7.4, and 7.8) was added to the three‐necked round‐bottom flask. Then, the solution of AB or HPB@Lip@AB (both containing 20 mg AB) was quickly dropped into the flask and gently stirred. At the pre‐determined time points, the volume of generated H_2_ gas was measured by the gas burette.

To detect the dissolved H_2_ content in solutions, AB or HPB@Lip@AB (both containing 20 mg AB) were added to a sealed 50 mL centrifuge tube. Then, 40 mL PBS (pH = 5.6, 6.2, 6.8, 7.2, 7.4, and 7.8) was added, and the H_2_ concentration was measured by a H_2_ detector.

### 5.4 SOD‐Like Activity

A modified NBT (S19048; Yuanye Biotechnology Co., Ltd., Shanghai, China) reduction method was applied to evaluate the SOD‐like activity. Typically, different amounts of HPB@Lip@AB (or the corresponding amounts of HPB, HPB@Lip, and AB) were added into a PBS solution, which contained methionine (39 mm), NBT (100 µm), riboflavin (100 µm; 013348991; Adamas‐Beta Co., Ltd.), and EDTA (1 mm). Then, the mixtures were placed in a 96‐well plate and illuminated at 5000 lux for 20 min under a lamp. The absorbance values before and after irradiation were measured at 560 nm using a multifunctional microplate reader (Model 550, Bio‐Rad, Hercules, CA, USA). The sample without HPB@Lip@AB was set as the control. The·O_2_^−^ inhibition rate was calculated according to Equation 3.

(3) $$\mathrm{Inhibition}\;\mathrm{ratio}\;\left(\%\right)=1-\frac{A_{1}-A_{0}}{A_{C1}-A_{C0}}\times 100$$

where A_0_ and A_1_ were the absorbance values of the sample before and after irradiation. A_C0_ and A_C1_ were the absorbance values of the control group before and after irradiation.

### 5.5 POD‐Like Activity

The POD‐like activity was measured using TMB (S19125; Yuanye Biotechnology Co., Ltd.) as an indicator. Briefly, 1 mL TMB solution (15 mg/mL in DMSO), 1 mL H_2_O_2_ (013397871; Adamas‐Beta Co., Ltd.) solution (200 mm), and 3 mL HAc‐NaAc buffer (50 mm, pH = 4) were mixed together as the reaction solution. Subsequently, 100 µL HPB@Lip@AB (or the corresponding amounts of HPB, HPB@Lip, and AB) and 100 µL reaction solution were added to a 96‐well plate. The absorbance values of oxidized TMB at 650 nm were measured at 0 and 20 min.

### 5.6 CAT‐Like Activity

The CAT‐like activity was evaluated by detecting the generated oxygen concentration. Briefly, different amounts of HPB@Lip@AB (or the corresponding amounts of HPB, HPB@Lip, and AB) were added to a PBS solution (pH = 7.4, containing 2.5% H_2_O_2_) at room temperature. After 5 min of H_2_O_2_ decomposition, the concentration of dissolved oxygen was measured by a dissolved oxygen meter (DO‐9100, Jiyi, China).

### 5.7 ·OH Scavenging Capacity

The ·OH scavenging capacity was detected using salicylic acid as the probe, which could be oxidized to purple 2,3‐dihydroxybenzoic acid by ·OH. Here, the Fenton reaction was used to produce ·OH: Fe^2+^ + H_2_O_2_ → Fe^3+^ + OH^−^ + ·OH. First, a mixed solution containing ferrous sulfate (FeSO_4_; 10 mm; 014348306; Adamas‐Beta Co., Ltd.) and H_2_O_2_ (10 mm) was reacted for 10 min at 37°C to produce ·OH. Then, 60 µL reaction solution was mixed with 100 µL HPB@Lip@AB solution (or the corresponding amounts of HPB, HPB@Lip, and AB) and reacted for another 30 min. At last, 40 µL salicylic acid (10 mm in ethanol) was added and maintained for 10 min. The absorbance value at 510 nm was measured. The sample without HPB@Lip@AB was set as the control. The ·OH scavenging rate was calculated according to Equation (4).

(4) $$\cdot \mathrm{OH}\;\mathrm{scavenging}\;\mathrm{rate}\left(\%\right)=1-\frac{A_{1}-A_{0}}{A_{C1}-A_{C0}}\times 100$$

where A_0_ and A_1_ were the absorbance values of the sample without and with salicylic acid. A_C0_ and A_C1_ were the absorbance values of the control group without and with salicylic acid.

### 5.8 ABTS·^+^ Scavenging Capacity

First, the stock solution was prepared by mixing ABTS (BD01314166; Bidepharmatech Co., Ltd.) solution (7.4 mm in ethanol) and K_2_S_2_O_8_ solution (2.5 mm in water) with the ratio of 1:1 (v/v). The stock solution was reacted for 12 h in the dark to produce ABTS·^+^, and then diluted with ethanol until an absorbance of 0.7 at 734 nm. Subsequently, the diluted solution was mixed with HPB@Lip@AB solution (or the corresponding amounts of HPB, HPB@Lip, and AB) at a ratio of 1:1 (v/v). The absorbance values at 734 nm were measured at 0 and 30 min. The sample without HPB@Lip@AB was set as the control. The ABTS·^+^ scavenging rate was calculated according to Equation (5)

(5) $$\text{ABTS}\cdot^{+}\;\text{scavenging rate}\left(\%\right)=1-\frac{A_{1}-A_{0}}{A_{\text{C1}}-A_{\text{C0}}}\times 100$$

where A_0_ and A_1_ were the absorbance values of the sample at 0 min and 30 min. A_C0_ and A_C1_ were the absorbance values of the control group at 0 and 30 min.

### 5.9 DPPH·Scavenging Capacity

First, 8 mg DPPH· (D807297; Heowns biochemical Co., Ltd., Tianjin, China) was dissolved in 50 mL ethanol to obtain the working solution. Then HPB@Lip@AB solution (or the corresponding amounts of HPB, HPB@Lip, and AB) was mixed with DPPH· working solution at a ratio of 1:1 (v/v), and incubated in the dark for 2 h at 37°C. Finally, the absorbance values at 517 nm were measured at 0 and 2 h. The sample without HPB@Lip@AB was set as the control. The DPPH· scavenging rate was calculated according to Equation (6).

(6) $$\mathrm{DPPH}\cdot \;\mathrm{scavenging}\;\mathrm{rate}\left(\%\right)=1-\frac{A_{1}-A_{0}}{A_{C1}-A_{C0}}\times 100$$

where A_0_ and A_1_ were the absorbance values of the sample at 0 h and 2 h. A_C0_ and A_C1_ were the absorbance values of the control group at 0 h and 2 h.

### 5.10 Nanomotor Motion Behavior

To visualize the nanomotor motion, C6‐labeled HPB@Lip and HPB@Lip@AB were first prepared by adding an appropriate amount of C6 during their synthesis. The motion behavior of the C6‐labeled nanomotors in simulated tear fluid was then observed using an inverted fluorescence microscope (Zeiss Axio Observer 7, Jena, Germany). Briefly, 1 µL of C6‐labeled HPB@Lip@AB (or HPB@Lip) solution and 10 µL of STF (containing 0, 1, or 10 mm H_2_O_2_) were added onto a glass slide, covered with a coverslip, and the motion was recorded by a CCD camera. Finally, the trajectories of at least 15 nanomotors in each group were analyzed using the Tracker software. The MSD, the D_eff,_ and the average speed (*v*) of nanomotors were calculated according to Equations ((7), (8), (9)).

(7) $$\text{MSD}\left(\Delta t\right)={\left[x\left(t+\Delta t\right)-x\left(t\right)\right]}^{2}+{\left[y\left(t+\Delta t\right)-y\left(t\right)\right]}^{2}$$

(8) $$D_{\mathrm{eff}\;}=\frac{\mathrm{MSD}}{4\Delta t}$$

(9) $$v=\frac{s}{t}$$

where x(*t*) and y(*t*) are the coordinates of the nanomotor at time t, Δ*t* is the time interval, and *s* is the travel distance in time *t* [79].

### 5.11 Cell Culture and Treatment

Human corneal epithelial (HCE‐T, CL‐0743) cells were obtained from Procell Life Science & Technology Co., Ltd. (Wuhan, China). Cells were maintained in DMEM/F‐12 medium (C11330500BT; Thermo Fisher Scientific) supplemented with 10% fetal bovine serum (A5256701; Gibco, Carlsbad, CA), 1% penicillin/streptomycin (C3024‐010; Vivacell, Shanghai, China), 5 µg/mL insulin (HY‐P0035; MedChemExpress, NJ, USA), and 10 ng/mL human epidermal growth factor (hEGF; PHG0314; Gibco), and incubated at 37°C under 5% CO_2_.

To establish an in vitro HS model in corneal epithelial cells, HCE‐T cells were cultured in hyperosmotic medium adjusted to 550 mOsm/L by supplementation with 94 mm NaCl. The cells were divided into the following treatment groups: a control group, an HS group, and three HS treatment groups treated with 200 µg/mL HPB@Lip, 20 µg/mL AB, or 200 µg/mL HPB@Lip@AB, respectively.

### 5.12 Cellular Uptake Assay

HCE‐T cells were seeded in 24‐well plates and cultured for 24 h. To simulate a hypertonic microenvironment, the cells were then pretreated with 550 mOsm/L medium for 24 h prior to the uptake assay. Subsequently, C6‐labeled HPB@Lip or HPB@Lip@AB was added to the wells and incubated for 0.5, 1, 2, and 4 h. Nuclei were stained with Hoechst 33342 (C1011; Beyotime Biotechnology, Shanghai, China). Fluorescence images were acquired using a DM6B Thunder imaging system (Leica Microsystems, Wetzlar, Germany), and cellular uptake was quantified using ImageJ (NIH, Bethesda, MD, USA).

### 5.13 Cytotoxicity Assays

Cytotoxicity was evaluated using the MTT assay (KGA1406‐5, Keygen Biotech, Nanjing, China). HCE‐T cells were seeded into 96‐well plates and incubated overnight. Subsequently, HPB@Lip, AB, or HPB@Lip@AB at various concentrations was added and incubated for 24 h. Thereafter, MTT reagent was added and incubated for 4 h. The resulting formazan crystals were dissolved with DMSO, and absorbance was measured at 570 nm using a SpectraMax 190 microplate reader (Molecular Devices, Sunnyvale, CA, USA).

### 5.14 TEM

For ultrastructural analysis of mitochondrial morphology, HCE‐T cells were processed for TEM. Cells were initially fixed with 2.5% glutaraldehyde in 0.1 m phosphate buffer (pH = 7.4) at 4°C for 4 h, then post‐fixed with 1% osmium tetroxide for 1.5 h. Samples were washed twice with cold PBS, dehydrated through a graded acetone series, and embedded in epoxide resin. Ultrathin sections were prepared, stained with uranyl acetate and lead citrate. TEM imaging was conducted with particular attention to mitochondrial cristae architecture and matrix density.

### 5.15 Measurement of ΔΨm

ΔΨm was assessed using the JC‐1 Assay Kit (C2006; Beyotime Biotechnology). Following treatments, cells were incubated with JC‐1 working solution (1:200 dilution) at 37°C for 20 min, then rinsed twice with assay buffer. Dual‐emission fluorescence images were captured on a DM6B Thunder imaging system (Leica), with quantification via ImageJ (NIH).

### 5.16 DHE and MitoSOX Staining

DHE and MitoSOX staining were performed as previously described [80]. Eyes were rapidly enucleated, embedded in OCT, and cryosectioned into 7 µm‐thick sections. Tissue sections were incubated with DHE (5 µm; S0064S; Beyotime Biotechnology) or MitoSOX (5 µm; M36008; Invitrogen, Carlsbad, CA, USA) for 30 min at 37°C. HCE‐T cells were stained with DHE (1 µm) or MitoSOX (1 µm) under identical conditions. Fluorescent images were acquired using a DM6B Thunder imaging system (Leica), with quantification via ImageJ (NIH).

### 5.17 Immunofluorescence

HCE‐T cell cultures on a 24‐well chamber slide were chemically fixed with freshly prepared 4% paraformaldehyde for 15 min, permeabilized with 0.1% Triton X‐100 for 15 min, blocked with 3% BSA for 30 min at room temperature and incubated with anti‐8‐OHdG (1:200 dilution, ab62623; Abcam, Cambridge, UK), anti‐LAMP1, anti‐SOD2, anti‐NLRP3, anti‐Caspase‐1, anti‐IL‐1β, and anti‐ALDH1L2 (1:200 dilution, 21997‐1‐AP, 66474‐1‐Ig, 27458‐1‐AP, 22915‐1‐AP, 226048‐1‐AP, 21391‐1‐AP; Proteintech, Rosemont, IL, USA) at 4°C overnight. Then cells were mounted with DAPI‐containing Mounting Medium (S2110; Solarbio, Beijing, China). Images were acquired by a DM6B Thunder imaging system (Leica), with quantification via ImageJ (NIH).

### 5.18 Measurement of ATP Content

A firefly luciferase ATP assay kit (S0026; Beyotime Biotechnology) was used to quantify ATP concentrations in HCE‐T cells, following the manufacturer's guidelines. Cell suspensions were combined with ATP detection working solution in white 96‐well plates. Standard curves were constructed in parallel, while protein concentrations across experimental groups were quantified via Bradford protein assay. ATP measurements were normalized to nmol/mg protein.

### 5.19 TUNEL Staining

HCE‐T cell cultures on 24‐well chamber slides were fixed with freshly prepared 4% paraformaldehyde for 15 min and permeabilized with 0.1% Triton X‐100 for 15 min, followed by staining using a TUNEL assay kit (C1090; Beyotime Biotechnology) according to the manufacturer's instructions. Images were captured with a Leica DM6B Thunder imaging system and quantified via ImageJ (NIH).

### 5.20 Western Blot

HCE‐T cells were lysed in RIPA buffer (P0013B; Beyotime Biotechnology) containing protease inhibitors (SJ‐MK0001‐A; SparkJade, Jinan, China) and phosphatase inhibitors (SJ‐MK0002‐A; SparkJade). Protein concentrations were quantified by BCA assay (SK1070; Coolaber, Beijing, China), with equal amounts of protein resolved on 10% SDS‐PAGE and transferred to PVDF membranes (IPVH00010; Millipore, Billerica, MA, USA). After blocking with 5% non‐fat milk, the membranes were incubated with antibodies and observed by the ECL Chemiluminescence Kit (180‐5001; Tanon, Shanghai, China). Antibodies against ALDH1L2 (21391‐1‐AP), BAX (50599‐2‐Ig), Bcl‐2 (80313‐1‐RR), GAPDH (6004‐1‐Ig), Goat Anti‐Rabbit IgG (SA00001‐2) and Goat Anti‐Mouse IgG (SA00001‐1) were obtained from Proteintech. Protein bands were quantified with ImageJ (NIH).

### 5.21 qPCR

Total RNA was isolated from HCE‐T cells using RNAex Pro Reagent (AG21102; Accurate Biotechnology, Changsha, China). RNA purity was assessed by measuring the A260/A280 absorbance ratio (1.8‐2.0). Reverse transcription of 1 µg RNA was performed with HiScript II Reverse Transcriptase (AG11711; Accurate Biotechnology) following the manufacturer's protocol. Quantitative PCR amplification was performed on a QuantStudio 5 Real‐Time PCR System (A28140; Thermo Fisher Scientific) using RNAex Pro SYBR Green Master Mix (AG11701; Accurate Biotechnology). Primer sequences used in this study were as follows: NOX4: 5′‐CAACTGTTCCTGGCCTGACA‐3′ (forward) and 5′‐GCAACGTCAGCAGCATGTAGA‐3′ (reverse); IL‐1β: 5′‐CTTTCCCGTGGACCTTCCA‐3′ (forward) and 5′‐CTCGGAGCCTGTAGTGCAGTT‐3′ (reverse); GAPDH: 5′‐GCCACCCAGAAGACTGTGGAT‐3′ (forward) and 5′‐GGAAGGCCATGCCAGTGA‐3′ (reverse).

### 5.22 Animal Experiments

Male C57BL/6J mice were purchased from the Liaoning Changsheng Biotechnology Co., Ltd. (Benxi, China). All animal protocols received approval from the Ethics Committee of Dalian Medical University (XL2604070207) and were conducted in compliance with the NIH Guide for the Care and Use of Laboratory Animals.

Two different DED mouse models were established, including a scopolamine hydrobromide‐induced aqueous‐deficient DED model and a BAC‐induced evaporative DED model. For the scopolamine hydrobromide‐induced DED model, mice received subcutaneous injections of 1.5 mg/0.3 mL scopolamine hydrobromide (HY‐N0296A; MedChemExpress) three times daily for 7 consecutive days. Model establishment was confirmed by sodium fluorescein staining and tear secretion tests. After model induction, mice were randomly assigned to the indicated treatment groups and received topical administration (5 µL per eye) with saline, HPB@Lip (200 µg/mL), AB (20 µg/mL), or HPB@Lip@AB (100 or 200 µg/mL) twice daily for 14 consecutive days. For the therapeutic efficacy comparison experiments, 0.05% CsA was included as a clinically relevant positive control.

For the BAC‐induced evaporative DED model, mice received topical administration of 0.2% (w/v) BAC eye drops (5 µL per eye) twice daily for 5 consecutive days to establish the evaporative DED model. After BAC induction, mice were randomly assigned to the indicated treatment groups and received topical administration (5 µL per eye) of saline or HPB@Lip@AB (200 µg/mL) twice daily for 14 consecutive days.

### 5.23 Retention Evaluation of HPB@Lip@AB on the Ocular Surface

To evaluate the ocular surface retention of HPB@Lip@AB under DED conditions, we first established a DED model in mice. RhB‐labeled HPB@Lip and HPB@Lip@AB were first prepared by adding an appropriate amount of RhB during their synthesis. Following topical administration, the fluorescence signals on the ocular surface of DED mice were monitored using an IVIS Lumina imaging system at predetermined time points (0, 0.5, 1, 5, 10, 20, 40, and 60 min) with excitation/emission wavelengths of 535/580 nm.

To further visualize the corneal distribution and retention of the formulations, cryosection imaging was performed. RhB, RhB‐HPB@Lip, and RhB‐HPB@Lip@AB were topically applied to the corneal surfaces of DED mice. At designated time points (30, 45 s, 5, 10, and 20 min), mice were euthanized, and eyeballs were collected and embedded in OCT compound (4583; Sakura, CA, USA) followed by rapid freezing in liquid nitrogen. Frozen sections (7 µm thickness) were prepared using a cryostat. Fluorescence images were acquired using a DM6B Thunder imaging system (Leica).

### 5.24 Tear Secretion Assessment

Tear secretion was assessed with phenol red cotton threads (20220511; Jingming, Tianjin, China) according to the manufacturer's instructions. The threads were placed at the lateral one‐third portion of the lower eyelid conjunctival sac. The moistened thread length was recorded as millimeters after 15 s without anesthesia.

### 5.25 TBUT Measurement

TBUT was measured as previously outlined [81]. Briefly, a 10 µL sodium fluorescein solution was administered into the inferior conjunctival sac. Mice were allowed to blink three times to distribute the dye. Corneas were examined using a cobalt blue‐filtered slit‐lamp, recording the time between the last blink and the first corneal dark spot appearance. Three measurements per eye were averaged as TBUT.

### 5.26 Fluorescein Staining

Corneal epithelial damage was assessed via fluorescein staining after instillation of sodium fluorescein into the conjunctival sac, with observation under a cobalt blue‐filtered slit‐lamp microscope. The cornea was divided into four areas (nasal, temporal, superior, and inferior), and each area was scored as follows: 0 = no staining, 1 = slight punctate staining, 2 = obvious punctate or mild coalescent staining, 3 = distinct coalescent or slight patchy staining, and 4 = distinct patchy staining.

### 5.27 Histopathological Analysis

After the animals were sacrificed, the eyeballs and conjunctival tissues, as well as major organs including the heart, liver, spleen, lung, and kidney, were collected and fixed in 4% paraformaldehyde and embedded in paraffin, cut into sagittal sections (5 µm thick). Sections were stained with H&E staining for histology. TUNEL staining was performed with a kit (C1089; Beyotime Biotechnology). Immunofluorescence utilized primary antibodies against SOD2, 8‐OHdG, NLRP3, Caspase‐1, IL‐1β, and ALDH1L2 (1:200 dilution, 66474‐1‐Ig, 27458‐1‐AP, 22915‐1‐AP, 226048‐1‐AP, 21391‐1‐AP; Proteintech). Images were captured with a DM6B Thunder imaging system (Leica), with quantification via ImageJ (NIH).

### 5.28 Retinal Thickness Measurements

Retinal thickness measurements were performed as previously described [82, 83]. Briefly, four H&E‐stained retinal sections from each eye were randomly selected, with adjacent sections separated by at least 60 µm. Retinal thickness was measured at a distance of 500 µm from the optic nerve head (ONH) using ImageJ (NIH).

### 5.29 Ocular Surface Imaging

Ocular surface images were acquired using a stereomicroscope to evaluate ocular surface morphology and potential abnormalities after treatment. Mice were anesthetized before imaging, and images were captured under identical illumination and magnification conditions to ensure reliable comparison among groups.

### 5.30 Measurement of IOP

IOP was measured using rebound tonometry (TonoLab; iCare, Espoo, Finland) without anesthesia. For each eye, three consecutive measurements were performed, and the mean value was used for subsequent statistical analysis.

### 5.31 RNA Sequencing Analysis

Transcriptome profiling was performed on corneal tissues from DED model mice (DED vs DED + H_2_, n = 3 per group), and HS‐treated HCE‐T cells (HS vs HS + HPB@Lip@AB, n = 3 per group) using Illumina‐based RNA sequencing (RNA‐seq). Sequencing services were provided by Seqhealth Technology Co., Ltd. (Wuhan, China) for murine samples and MetWare Biotechnology Co., Ltd. (Wuhan, China) for cellular samples. DEGs were identified through DESeq2 v1.38.3 analysis with Benjamini‐Hochberg correction, applying thresholds of |log_2_FC| > 0.58 (*p* < 0.05) for murine data and |log_2_FC| > 1 (*p* < 0.05) for cellular data.

Mitochondria‐related genes were curated from MitoCarta 3.0 (https://www.broadinstitute.org/mitocarta). Mitochondria‐related DEGs (MitoDEGs) were defined as the intersection between DEGs and MitoCarta 3.0 entries, visualized through Venn diagrams generated using the Venn diagram tool from Bioinformatics & Evolutionary Genomics.

### 5.32 RNA Interference

Small interfering RNA (siRNA) was transfected into HCE‐T cells using siRNA‐mate plus transfection kit (G04026; Genepharma, Shanghai, China). Sequences of siRNA were as follows: ALDH1L2 sense: 5′‐GCACGGCUCUAUCAUUUAUTT‐3′, antisense: 5′‐AUAAAUGAUAGAGCCGUGCTT‐3′. A non‐targeting siRNA sequence served as a negative control. Briefly, the siRNA was dissolved in diethylpyrocarbonate (DEPC) water at a concentration of 20 µm. The siRNA was mixed with DMEM/F12 medium (C11330500BT; Thermo Fisher Scientific) and incubated at room temperature for 5 min. Cells were trypsinized, resuspended, and then mixed with the siRNA solution. The cell suspension was divided equally among six‐well plates. Each well contained 2 mL of culture medium, 4.5 µL siRNA‐mate plus, and 3.75 µL siRNA (75 pmol). The transfection time was 48 h, and knockdown efficiency was verified by western blot analysis.

### 5.33 Adenoviral Infection

Recombinant adenoviruses encoding ALDH1L2 overexpression construct (Ad‐ALDH1L2) or an empty vector control (Ad‐empty) were generated by Weizhen Bioscience Inc. (Jinan, China). At 24 h post‐seeding, cells were transduced with Ad‐empty (50 MOI) or Ad‐ALDH1L2 (50 MOI) for 24 h.

### 5.34 Statistical Analysis

Normally distributed data are presented as mean ± standard deviation (SD). Statistical analyses were performed using GraphPad Prism 8.0 software. Statistical significance was determined using one‐way or two‐way analysis of variance (ANOVA), followed by Tukey's or Šídák's multiple comparisons tests, as appropriate. For experiments involving repeated measurements over time, two‐way repeated measures ANOVA followed by Šídák's multiple comparisons test was applied. The following significance levels were used: ns, not significant; ^*^ *p* < 0.05, ^**^ *p* < 0.01, ^***^ *p* < 0.001, and ^****^ *p* < 0.0001.

## Author Contributions

**Jing Li**: conceptualization, methodology, software, data curation, investigation, visualization, writing – original draft, funding acquisition. **Xi Wan**: methodology, software, data curation, validation, visualization, writing – original draft, investigation. **Xinru Su**: methodology, software, data curation, visualization, validation. **Zechuan Li**: validation, methodology, investigation. **Yue Wang**: visualization, methodology, data curation. **Ying Wang**: methodology, software, data curation. **Ruofei Li**: methodology, software. **Huangzhao Wei**: resources, visualization, software. **Jianbin Zhang**: conceptualization, software, data curation, supervision, visualization, writing – review and editing, project administration, resources, methodology, writing – original draft, validation. **Shuai Wang**: conceptualization, investigation, funding acquisition, methodology, validation, visualization, writing – review and editing, formal analysis, project administration, supervision, resources.

## Conflicts of Interest

The authors declare that they have no competing financial interests. A patent related to the Prussian blue‐based composite material has been granted (Patent No. CN120324462B), with some authors of this manuscript listed as inventors.

## Supporting information




**Supporting File 1**: advs77681‐sup‐0001‐SuppMat.zip.

**Supporting File 2**: advs77681‐sup‐0002‐SuppMat.xlsx.

**Supporting File 3**: advs77681‐sup‐0003‐SuppMat.xlsx.

**Supporting File 4**: advs77681‐sup‐0004‐SuppMat.docx.

## Data Availability

The raw RNA‐seq data are available from the corresponding author upon reasonable request. All other data supporting the findings of this study are available in the article and its Supplementary Materials.
