# Supplementary material for: Self‐Propelled HPB@Lip@AB Nanomotors Ameliorate Dry Eye Disease
Source: Adv Sci (Weinh). 2026 Sep 22:e77681. Online ahead of print. doi: 10.1002/advs.77681 (PMC13598384; doi:10.1002/advs.77681)
Supplement: Supplementary file 1 [file ADVS-9999-e77681-s003.docx]

**Supporting Information**

**Title：Self-Propelled HPB@Lip@AB Nanomotors Ameliorate Dry Eye Disease**

Jing Li^1, 2^, Xi Wan^1^, Xinru Su^1^, Zechuan Li^1^, Yue Wang^2^, Ying Wang^2^, Ruofei Li^2^, Huangzhao Wei^3^, Jianbin Zhang^4, *^ & Shuai Wang^1, *^

^1^Department of Ophthalmology, Second Affiliated Hospital of Dalian Medical University, Dalian 116027, China

^2^Institute of Heart and Vascular Diseases, Second Affiliated Hospital of Dalian Medical University, Dalian 116027, China

^3^Dalian Institute of Chemical Physics, Chinese Academy of Sciences, Dalian 116027, China

^4^College of Pharmacy, Dalian Medical University, Dalian 116044, China

*Correspondence: Jianbin Zhang (zhangjb@dmu.edu.cn); Shuai Wang (wang.s@dmu.edu.cn)

Jing Li and Xi Wan contributed equally to this work.

**Supplementary Figures**

**Figure S1. Photograph showing aqueous dispersions of HPB, HPB@Lip, and HPB@Lip@AB.**

**Figure S2. High-resolution XPS spectra of HPB, HPB@Lip, and HPB@Lip@AB.** (**A**) C 1s, (**B**) Fe 2p, (**C**) Fe 3p, (**D**) N 1s, (**E**) O 1s, and (**F**) P 2p.

**Figure S3. Colloidal stability evaluation of HPB-based nanocomposites under different physiological conditions.** (**A-C**) Particle size changes of HPB (**A**), HPB@Lip (**B**), and HPB@Lip@AB (**C**) in water, PBS, STF, DMEM, and DMEM + FBS during 48 h incubation.

**Figure S4. Photographs showing the H₂ release behavior of HPB@Lip@AB.** Generation of H₂ bubbles under different pH solutions.

**Figure S5. Lysosome-associated intracellular localization of HPB@Lip@AB in HCE-T cells**. Representative images showing the partial colocalization of C6-labeled HPB@Lip@AB (green) with LAMP1-positive lysosome-associated regions (red) in HCE-T cells, Scale bar, 10 μm (n = 3).

**Figure S6. Photographs showing the multienzyme-like activity and antioxidant activity of HPB@Lip@AB.** (**A**) SOD-like activity of HPB, HPB@Lip, AB, and HPB@Lip@AB determined by the NBT assay. (**B**) CAT-like activity evaluated by the decomposition of H₂O₂ and oxygen generation. (**C**) POD-like activity measured using TMB oxidation. (**D**) ·OH scavenging activity determined by the salicylic acid assay. (**E**) ABTS·⁺ radical scavenging activity. (**F**) DPPH· radical scavenging activity. HPB@Lip@AB exhibited enhanced antioxidant performance by combining the enzyme-mimetic activity of HPB and the H₂-generating antioxidant function of AB.

**Figure S7.** **Cellular uptake of HPB@Lip and HPB@Lip@AB in HCE-T cells.** Quantification of intracellular fluorescence intensity of C6-labeled HPB@Lip and HPB@Lip@AB in HCE-T cells (n = 3; two-way ANOVA with Šídák’s multiple comparisons test). Data are presented as mean ± SD. ns, not significant.

**Figure S8. In vitro antioxidant effect of HPB@Lip@AB.** (**A** and **B**) Representative DHE fluorescence images of HCE-T cells, accompanied by statistical data, Scale bar, 25 μm (n = 6; one-way ANOVA with Tukey’s multiple comparisons test). (**C**) qPCR analysis of NOX4 mRNA levels in HCE-T cells (n = 3; one-way ANOVA with Tukey’s multiple comparisons test). (**D** and **E**) Representative immunofluorescence images of HCE-T cells stained with SOD2, accompanied by statistical data, Scale bar, 25 μm (n = 6; one-way ANOVA with Tukey’s multiple comparisons test). Data are presented as mean ± SD; ^****^P < 0.0001 vs. CON + PBS group; ^####^P < 0.0001 vs. HS + PBS group.

**Figure S9. In vitro anti‑inflammatory effect of HPB@Lip@AB.** (**A** and **B**) Representative immunofluorescence images of HCE‑T cells stained with NLRP3, accompanied by statistical data, Scale bar, 25 μm (n = 6; one-way ANOVA with Tukey’s multiple comparisons test). (**C** and **D**) Representative immunofluorescence images of HCE‑T cells stained with Caspase-1, accompanied by statistical data, Scale bar, 25 μm (n = 6; one-way ANOVA with Tukey’s multiple comparisons test). (**E** and **F**) Representative immunofluorescence images of HCE‑T cells stained with IL-1β, accompanied by statistical data, Scale bar, 25 μm (n = 6; one-way ANOVA with Tukey’s multiple comparisons test). (**G**) qPCR analysis of IL-1β mRNA levels in HCE‑T cells (n = 3; one-way ANOVA with Tukey’s multiple comparisons test). Data are presented as mean ± SD; ^****^P < 0.0001 vs. CON + PBS group; ^###^P < 0.001 and ^####^P < 0.0001 vs. HS + PBS group.

**Figure S10. In vivo comparison of the therapeutic efficacy of HPB@Lip@AB and 0.05% cyclosporine A (CsA).** (**A**) Schematic illustration of the DED model and treatment regimen. DED was induced by subcutaneous injection of scopolamine hydrobromide (1.5 mg/0.3 mL, three times daily) for 7 days (day −7 to day 0). After model establishment on day 0, mice received bilateral topical administration (5 μL) of saline, 200 μg/mL HPB@Lip@AB, or 0.05% CsA for 14 days (day 0 to day 14). Ocular surface assessments were performed on days 0, 7, and 14. (**B**) Representative corneal fluorescein staining images. (**C-E**) Quantitative analysis of corneal fluorescein staining scores, tear secretion evaluated by phenol red thread test, and tear film break-up time (TBUT), respectively (n = 6; two-way ANOVA with Tukey’s multiple comparisons test). (**F**) Representative H&E staining images of corneal tissues showing epithelial and stromal structures, Scale bar, 50 μm. (**G**) Quantitative analysis of the corneal epithelial thickness (n = 6; one-way ANOVA with Tukey’s multiple comparisons test). Data are presented as mean ± SD; ^**^P < 0.01 and ^****^P < 0.0001 vs. DED + Saline group; ^#^P < 0.05, ^##^P < 0.01, and ns vs. DED + 0.05% CsA group. Epi, corneal epithelium; Stroma, corneal stroma; Endo, corneal endothelium.

**Figure S11. In vivo therapeutic efficacy of HPB@Lip@AB in a benzalkonium chloride (BAC)-induced evaporative DED model.** (**A**) Schematic illustration of the evaporative DED model and treatment regimen. DED was induced by topical administration of BAC for 5 consecutive days. From day 0, mice received bilateral topical administration (5 μL) of saline or 200 μg/mL HPB@Lip@AB for 14 consecutive days. Ocular surface assessments were performed on days 0, 7, and 14. (**B**) Representative corneal fluorescein staining images. (**C-E**) Quantitative analysis of corneal fluorescein staining scores, tear secretion evaluated by phenol red thread test, and TBUT, respectively (n = 6; two-way ANOVA with Tukey’s multiple comparisons test). (**F**) Representative H&E staining images of corneal tissues showing epithelial and stromal structures, Scale bar, 50 μm. (**G**) Quantitative analysis of corneal epithelial thickness. (n = 6; one-way ANOVA with Tukey’s multiple comparisons test) Data are presented as mean ± SD; ^****^P < 0.0001 vs. CON + Saline group; ^###^P < 0.001 and ^####^P < 0.0001 vs. DED + Saline group. Epi, corneal epithelium; Stroma, corneal stroma; Endo, corneal endothelium.

**Figure S12. In vivo anti-inflammatory effect of HPB@Lip@AB.** (**A**) Representative immunofluorescence images of corneal tissues stained with NLRP3, accompanied by statistical data, Scale bar, 50 μm (n = 6; one-way ANOVA with Tukey’s multiple comparisons test). (**B**) Representative immunofluorescence images of corneal tissues stained with Caspase-1, accompanied by statistical data, Scale bar, 50 μm (n = 6; one-way ANOVA with Tukey’s multiple comparisons test). (**C**) Representative immunofluorescence images of corneal tissues stained with IL-1β, accompanied by statistical data, Scale bar, 50 μm (n = 6; one-way ANOVA with Tukey’s multiple comparisons test). Data are presented as mean ± SD; ^****^P < 0.0001 vs. CON + Saline group; ^####^P < 0.0001 vs. DED + Saline group. Epi, corneal epithelium; Stroma, corneal stroma; Endo, corneal endothelium.

**Figure S13. Ocular and systemic biosafety evaluation and 30-day ocular tolerance of HPB@Lip@AB.** (**A**) Schematic illustration of the experimental design for systemic and ocular biosafety evaluation. Healthy mice received topical administration of PBS, HPB@Lip, AB, or HPB@Lip@AB twice daily for 14 consecutive days. **(B)** Quantitative analysis of retinal thickness after 14 days of topical administration (n = 3; one-way ANOVA with Tukey’s multiple comparisons test). **(C)** Representative H&E staining images of conjunctival, retinal, heart, liver, spleen, lung, and kidney tissues after 14 consecutive days of topical administration (n = 3). **(D)** Schematic illustration of the experimental design for long-term ocular tolerance evaluation. Healthy mice received topical administration of PBS or HPB@Lip@AB twice daily for 30 consecutive days. **(E)** Changes in intraocular pressure (IOP) during the 30-day administration period (n = 3; two-way repeated measures ANOVA). (**F**) Representative ocular surface images, corneal fluorescein staining images, and H&E staining images of conjunctival and retinal tissues after 30 consecutive days of topical administration (n = 3). **(G)** Quantitative analysis of retinal thickness after 30-day administration (n = 3; one-way ANOVA with Tukey’s multiple comparisons test). Data are presented as mean ± SD; ns, not significant vs. PBS group.

**Figure S14. H_2_ via ALDH1L2 regulates oxidative stress in vitro.** (**A** and **B**) Representative DHE fluorescence images of HCE-T cells, accompanied by statistical data, Scale bar, 25 μm (n = 6; one-way ANOVA with Tukey’s multiple comparisons test). (**C**) qPCR analysis of NOX4 mRNA levels in HCE-T cells (n = 3; one-way ANOVA with Tukey’s multiple comparisons test). (**D** and **E**) Representative immunofluorescence images of HCE-T cells stained with SOD2, accompanied by statistical data, Scale bar, 25 μm (n = 6; one-way ANOVA with Tukey’s multiple comparisons test). Data are presented as mean ± SD; ^*^P < 0.05, ^**^P < 0.01, ^***^P < 0.001, and ^****^P < 0.0001 vs. HS + PBS group; ^####^P < 0.0001 vs. HS + HPB@Lip@AB group.

**Figure S15. H_2_ via ALDH1L2 regulates inflammation in vitro.** (**A** and **B**) Representative immunofluorescence images of HCE-T cells stained with NLRP3, accompanied by statistical data, Scale bar, 25 μm (n = 6; one-way ANOVA with Tukey’s multiple comparisons test). (**C** and **D**) Representative immunofluorescence images of HCE-T cells stained with Caspase-1, accompanied by statistical data, Scale bar, 25 μm (n = 6; one-way ANOVA with Tukey’s multiple comparisons test). (**E** and **F**) Representative immunofluorescence images of HCE-T cells stained with IL-1β, accompanied by statistical data, Scale bar, 25 μm (n = 6; one-way ANOVA with Tukey’s multiple comparisons test). (**G**) qPCR analysis of IL-1β mRNA levels in HCE-T cells (n = 3; one-way ANOVA with Tukey’s multiple comparisons test). Data are presented as mean ± SD; ^*^P < 0.05, ^**^P < 0.01, and ^****^P < 0.0001 vs. HS + PBS group; ^#^P < 0.05, and ^####^P < 0.0001 vs. HS + HPB@Lip@AB group.
